# Comparative Genomic Analysis of *Drosophila melanogaster* and Vector Mosquito Developmental Genes

**DOI:** 10.1371/journal.pone.0021504

**Published:** 2011-07-06

**Authors:** Susanta K. Behura, Morgan Haugen, Ellen Flannery, Joseph Sarro, Charles R. Tessier, David W. Severson, Molly Duman-Scheel

**Affiliations:** 1 Department of Biological Sciences and Eck Institute for Global Health, University of Notre Dame, Notre Dame, Indiana, United States of America; 2 Department of Medical and Molecular Genetics, Indiana University School of Medicine, South Bend, Indiana, United States of America; Universidade Federal do Rio de Janeiro, Brazil

## Abstract

Genome sequencing projects have presented the opportunity for analysis of developmental genes in three vector mosquito species: *Aedes aegypti*, *Culex quinquefasciatus*, and *Anopheles gambiae*. A comparative genomic analysis of developmental genes in *Drosophila melanogaster* and these three important vectors of human disease was performed in this investigation. While the study was comprehensive, special emphasis centered on genes that 1) are components of developmental signaling pathways, 2) regulate fundamental developmental processes, 3) are critical for the development of tissues of vector importance, 4) function in developmental processes known to have diverged within insects, and 5) encode microRNAs (miRNAs) that regulate developmental transcripts in *Drosophila*. While most fruit fly developmental genes are conserved in the three vector mosquito species, several genes known to be critical for *Drosophila* development were not identified in one or more mosquito genomes. In other cases, mosquito lineage-specific gene gains with respect to *D. melanogaster* were noted. Sequence analyses also revealed that numerous repetitive sequences are a common structural feature of *Drosophila* and mosquito developmental genes. Finally, analysis of predicted miRNA binding sites in fruit fly and mosquito developmental genes suggests that the repertoire of developmental genes targeted by miRNAs is species-specific. The results of this study provide insight into the evolution of developmental genes and processes in dipterans and other arthropods, serve as a resource for those pursuing analysis of mosquito development, and will promote the design and refinement of functional analysis experiments.

## Introduction

Blood feeding mosquitoes, including *Aedes aegypti* (dengue and yellow fever vector), *Culex quinquefasciatus* (lymphatic filariasis and West Nile vector), and *Anopheles gambiae* (malaria vector), transmit many of the world's deadliest diseases. Detailed comparative analyses of mosquito developmental genetics will undoubtedly yield important advancements in the study of insect evolution of development and may reveal novel opportunities for vector control. Although the genomes of these three important insect vectors of human disease have been sequenced, little is known about genes that regulate development of these or other mosquito species. Unfortunately, very few descriptions of mosquito development presently exist. *A. vexans* is likely the most carefully described Aedine mosquito species [1]. However, the genome of *A. vexans* has not yet been sequenced, and the function of developmental genes have yet to be assessed in this species. Of the mosquito species for which genome sequences are available [2], [3], [4], both *A. aegypti* embryonic development [5], [6], [7] and *C. quinquefasciatus* [8] development have been staged. However, expression of only a handful of developmental genes have been characterized in these or other vector mosquitoes [9], [10], [11], [12], [13], [14], [15], [16], [17], [18].

The current lack of developmental genetic studies in mosquitoes is in part due to the technical challenges encountered by those who have attempted to analyze mosquito development. For example, the chorion and serosal cuticle of *A. aegypti*, which serve as barriers to fixatives, probes, and antibodies, have made working with this species a challenge in the past [7]. We recently published procedures for egg collection, tissue preparation, gene and protein expression, and RNAi-mediated functional analysis of developmental genes in *A. aegypti* [7], [17], [19], [20], [21], [22]. These and other previously developed methodologies [9], [11], in combination with the three mosquito genome sequences, are facilitating developmental studies in vector mosquitoes. Moreover, these advancements present an excellent opportunity to extend studies of the evolution of developmental genes and pathways in insects. With methodologies for comparative analysis of mosquito developmental genetics in hand, an existing challenge is to utilize the wealth of information provided by the genome projects [2], [3], [4] to identify changes in developmental genes that may underlie the morphological and biological differences observed among these insects.

In recent years, evolutionary developmental biologists have applied knowledge of developmental genetics in *D. melanogaster*, a well-characterized genetic model organism, to better understand development of other arthropods. Embryonic development of mosquitoes is superficially comparable to that of *Drosophila* in that mosquitoes, like fruit flies, are holometabolous, long germ band insects [23]. Comparison *of D. melanogaster* and *A. aegypti* development suggests that major developmental events are generally well conserved between the two species [7]. However, while knowledge of fruit fly development can serve as a springboard for developmental studies in non-model arthropods like mosquitoes, the *D. melanogaster* and mosquito insect lineages separated 260 million years ago (mya) (discussed in [4]), and it is anticipated that detailed comparative analyses will uncover many divergent developmental processes among these insects. Likewise, as discussed by Arensburger et al. [4], the Anopheline and Culicine lineages separated 145–200 mya, and the *C. quinquefasciatus* and *A. aegypti* lineages diverged 52–54 mya. Thus, it is also likely that one will encounter numerous differences in the development of these vector mosquito species.

In this investigation, we performed a genome-wide comparison of developmental genes in *D. melanogaster*, *A. aegypti*, *C. quinquefasciatus*, and *A. gambiae*. Based on similar genome-wide comparisons in other insect species such as the honey bee *Apis mellifera* [24] and pea aphid *Acyrthosiphon pisum* [25], it was hypothesized that although many *D. melanogaster* developmental genes will be highly conserved among the three vector mosquito species, lineage specific duplications, expansions, and losses underlying basic biological differences between these distantly related insects would be observed. To assess this, ortholog assignments for *D. melanogaster* developmental genes in the three mosquito genomes were compiled. Following completion of the *A. gambiae* genome project, Zdobnov et al. [26] prepared a list of *Drosophila* developmental genes that have orthologs in *A. gambiae*. An expanded and updated list of developmental gene ortholog assignments (including relevant gene identification numbers) for *A. gambiae* is included here. Furthermore, with the completion of two Culicine genome sequences, it is now possible to gain more insight into the evolutionary dynamics of developmental genes in mosquitoes. Ortholog assignments for *A. aegypti* and *C. quinquefasciatus* are therefore included in this investigation.

While this developmental gene analysis is inclusive, particular attention is focused on genes that 1) are components of conserved developmental signaling pathways (canonical and non-canonical Wnt, Notch, Jak-STAT, Hedgehog, Receptor Tyrosine Kinase, and TGFβ), 2) regulate fundamental developmental processes (axis patterning, segmentation and segmental patterning, germline development, neurogenesis, and apoptosis), 3) are critical for the development of tissues known to be vital to mosquito host location and the spread of infection (salivary gland, olfactory system, and larval cuticle), 4) function in developmental processes known to have diverged within insects (head development, sex determination, dosage compensation, and egg diapause, and 5) encode miRNAs that regulate developmental transcripts in *D. melanogaster*.

## Results and Discussion

### Components of conserved developmental signaling pathways

The components of several developmental signaling pathways are highly conserved in both vertebrate and invertebrate species [27]. It is anticipated that many of these signaling cascades, which are employed in a variety of developmental processes, will be well conserved in vector mosquitoes. Such was the case in comprehensive surveys of both the *A. mellifera* and *A. pisum* genomes [24], [25]. In this investigation, components of the Wnt, non-canonical Wnt, Notch, Jak-STAT, Hedgehog, Receptor Tyrosine Kinase, and TGF*β* signal transduction cascades were examined. Findings are summarized below, and ortholog assignments for the genes discussed are provided in Table S1. Information regarding mosquito lineage specific absences or gains of signaling pathway genes (with respect to *D. melanogaster*) is presented in Table 1.

**Table 1:** Analysis of genes encoding components of major developmental signaling pathways.

Pathway Component	*Aae*	*Cqu*	*Aga*
**Canonical Wnt**			
*APC*	0	1	1
*fz1*	4	3	2
*pan/TCF*	2	3	2
*wg*	2	1	1
*Wnt2*	2	1	1
*Wnt4*	2	1	1
*WntD*	0	1	0
**Non-canonical Wnt**			
*dgo*	1	3	0
**Notch**			
*Dl*	1	0	1
*N*	1	? see text	1
*Ser*	1	2	1
**JAK-STAT**			
*os*	0	0	0
*Stat92E*	1	3	2
**Hh**			
*slmb*	1	2	1
**EGF**			
*argos*	0	0	0
*Cbl*	2	2	1
*grk*	0	0	0
*rho*	0	0	0
*S*	1	1	0
**FGF**			
*bnl*	0	2	1
*htl*	2	2	1
**Ras**			
*hep*	1	2	1
*nemo*	2	1	1
*rl*	2	1	1
**TGF-beta**			
*Actbeta*	0	0	1
*Dad*	0	0	0
*sax*	2	1	1
*scw*	0	0	0
*Smox*	1	2	1
*tkv*	2	1	1

The number of orthologs encoding various Wnt, non-canonical Wnt, Notch, Jak-STAT, Hedgehog, Receptor tyrosine kinase, and TGF*β* signaling pathway components are indicated for each of the three mosquito species. Numbers refer to the number of orthologous sequences present in the three mosquito genomes for each *D. melanogaster* gene indicated at left. Results are reported only for genes in which the number of orthologous sequences varies between *D. melanogaster* and at least one of the mosquito species. Although the pathway components are generally very well conserved, changes in the number of orthologous sequences for several genes encoding components of the indicated signaling pathways (most notably Wnt, Notch, and FGF) were observed.

#### *Wnt*

**Figure 1:**
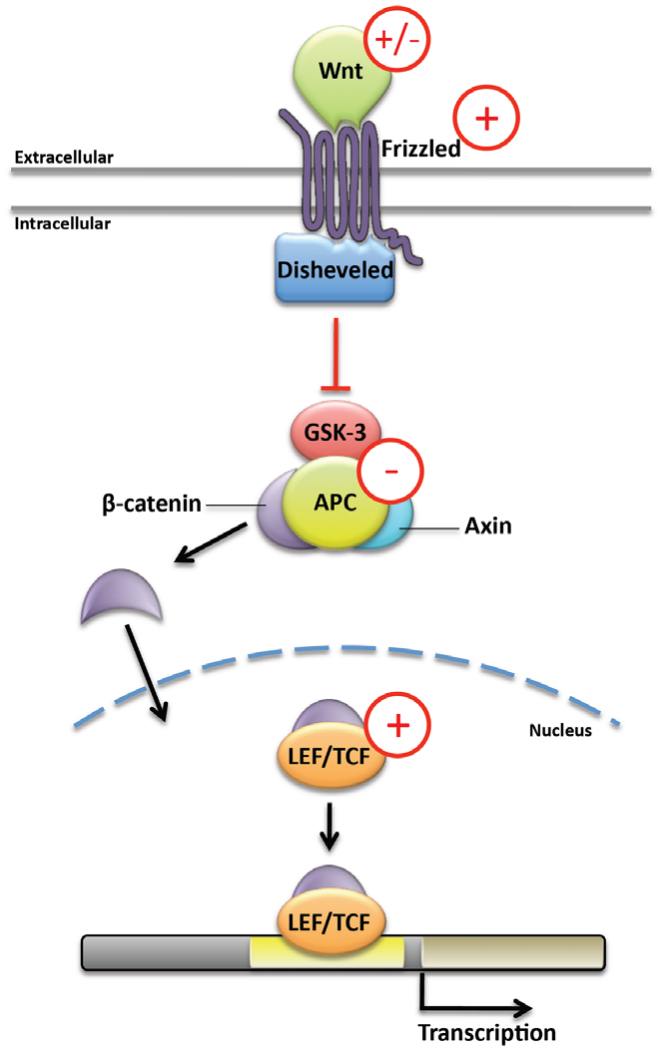
Wnt Signaling Pathway Components in Mosquitoes. The canonical Wnt pathway is summarized in this figure. Wnt binds its receptor Fz, which activates Dsh, an inhibitor of GSK-3. GSK-3 normally prevents dissociation of β-catenin from APC; when GSK-3 is inhibited, β-catenin enters the nucleus and regulates transcription (reviewed in [28]). Analysis of pathway members uncovered mosquito lineage specific changes in the number of orthologous sequences for genes encoding various Wnt pathway components in mosquitoes. With respect to *D. melanogaster*, mosquito lineage specific gene absences (−), as well as increases in the number of orthologues (+) were noted for particular pathway members. Details are provided in the text.

In *D. melanogaster*, Wnt signaling regulates a variety of developmental processes, ranging from segmentation and nervous system development to organogenesis and imaginal disc development [28]. Wnt signaling pathway components were examined in the three vector mosquito species, and findings are summarized in Figure 1. Members of the *Wnt* gene family encode ligands for this pathway. *D. melanogaster* and vector mosquitoes share *wingless (wg, Wnt-1)*, *Wnt-2*, *Wnt-4*, *Wnt-5*, *Wnt-6*, and *Wnt-10* orthologs. *Wnts 7* and *9*, which are found in the fruit fly, were not identified in any of the mosquito genomes. The *D. melanogaster Wnt D* gene was identified only in *C. quinquefasciatus*. An additional *Wnt* ortholog most closely resembling *Wnt2* and *Wnt4*, but with no apparent *D. melanogaster* ortholog, was identified in all three vector mosquitoes. *Wnts 11*, *16*, and *A*, all of which were identified in the *A. pisum* genome [25], were not found in mosquitoes. Ancestrally, bilaterian animals had 11 Wnt genes [29], suggesting that vector mosquitoes and *Drosophila* have differentially lost *Wnt* genes, as was the conclusion drawn from comparisons between the *A. mellifera* and *Drosophila* genomes [24]. Despite these apparent losses, two *wg*, *Wnt2*, and *Wnt4* genes were identified in *A. aegypti*, suggesting that duplications may have occurred in this lineage.

**Figure 2:**
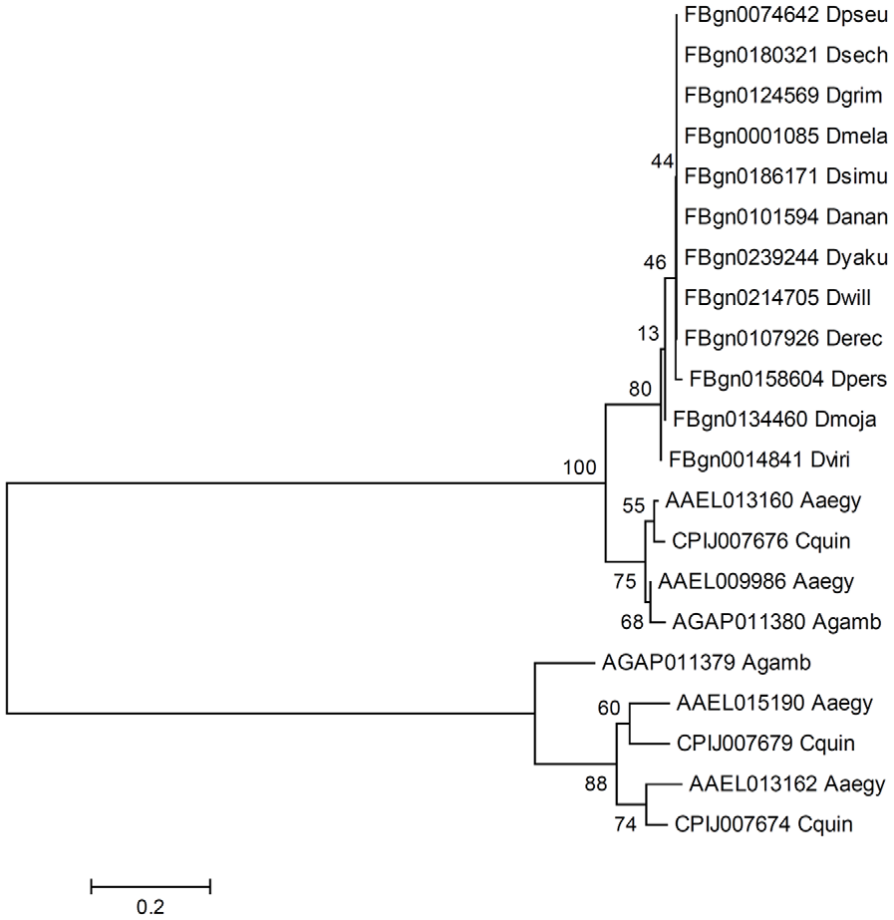
Evolutionary relationships of *fz* genes. Relationships among orthologous mosquito and *Drosophila* Fz (Fz-1) proteins were inferred using the Neighbor-Joining method. The gene ID and the species name (5 letters) are shown for the orthologs. The optimal tree (the sum of branch length = 2.675) is shown. The percentage values of replicate trees in which the associated taxa clustered together following bootstrap testing (1000 replicates) are shown next to the branches. The tree is drawn to scale (shown below the tree), with branch lengths in the same units as those of the evolutionary distances used to infer the phylogeny. The distance scale is in units of the number of amino acid substitutions per site. The phylogenetic branching suggests that there could be an ancestral as well as a modern origin of mosquito *fz* genes in relation to *Drosophila*.

Orthologs of the *frizzled (fz)* family of Wnt receptors were also identified. Single *fz2*, *3*, and *4* orthologs were identified in all three mosquitoes. An interesting expansion of *fz (fz1)* was observed in all three mosquitoes. Four *fz* orthologs were found in *A. aegypti*, three in *C. quinquefasciatus*, and two in *A. gambiae*. The phylogenetic branching pattern of fruit fly and mosquito *fz* genes (Figure 2) suggests that there could be an ancestral as well as a modern origin of mosquito *fz* genes in relation to *Drosophila*. The most common ancestor of *fz* genes from which these branches might have diverged shows a single phylogenetic grouping of ancestral mosquito and fruit fly *fz* genes. The modern *fz* genes are specific to the mosquitoes only and are not present in fruit flies. If these neo *fz* genes have retained *fz* functionality in mosquitoes, it may suggest a functional enhancement of Wnt receptors in mosquitoes that may be either novel or complementary to the ancestral receptor functionalities.

Most major downstream components of the canonical Wnt pathway were identified in all three vector mosquitoes (Figure 1). Single *disheveled*, *arrow*, *axin*, *shaggy/GSK3*, and *armadillo/β-catenin* genes were identified in each species. However, no ortholog of *adenomatous polyposis coli (APC)*, which encodes a regulator of β-catenin levels, was found in *A. aegypti*. Increased numbers of several canonical Wnt pathway members were also noted. For example, two orthologs of the transcriptional regulator *Pangolin/TCF* were identified in both *A. aegypti* and *A. gambiae*, while three were found in *C. quinquefasciatus*.

Core non-canonical Wnt/planar cell polarity (PCP) signaling components were also examined. PCP signaling regulates the coordinated orientation of cells and cellular structures along an axis within the plane of an epithelial surface. Core PCP signaling genes become localized to either the distal or proximal ends of the cells, where they are believed to communicate tissue polarity information to neighboring cells. Distally localized Fz binds and recruits Dsh, which in turn binds the ankyrin-repeat protein Diego (Dgo) and recruits it to the distal complex. The transmembrane protein Van Gogh is localized at the proximal end of the cell, where it recruits the LIM domain protein Prickle. Starry Night/Flamingo, a protocadherin, is enriched at both the proximal and distal cell junctions [30]. In general, genes encoding core PCP pathway components were very well conserved in vector mosquitoes, with the exception of *dgo*. Although a single *dgo* gene was identified in *A. aegypti*, no *dgo* gene was found in *A. gambiae*, while three *dgo* genes were found in *C. quinquefasciatus*. Given that *Drosophila* PCP signaling regulates organization of the surface bristles on the body, hairs on the wing, and photoreceptors of the eye [31], it would be interesting to examine how changes in the mosquito PCP pathway have impacted the functions of this signaling cascade during mosquito development. Such analyses are important, as the PCP pathway has not yet been studied in an arthropod evolutionary developmental context.

#### Notch (N)

N signaling regulates cell fate determination in flies, perhaps most notably during neurogenesis, though its function is required in many tissues [32]. Although single *N* genes were identified in both *A. aegypti* and *A. gambiae*, no ortholog of the *D. melanogaster N* gene was found in *C. quinquefasciatus* [33]. Two genes are referred to as *“N”* in Vectorbase [34]: CPIJ005570, which bears considerable sequence similarity to *D. melanogaster* N, and CPIJ011346, which bears much less sequence similarity to *Drosophila* N. A third gene CPIJ005569, is referred to as *“Neurogenic locus N;”* although the predicted protein product bears sequence similarity to *Drosophila* N, it is quite a bit shorter in length. Furthermore, none of these genes are orthologous to the *A. aegypti* or *A. gambiae N* genes. The status of any of these genes as *N* orthologs is therefore uncertain. Similarly, *Delta (Dl)*, which encodes a N ligand, was found in *A. aegypti* and *A. gambiae*, but not *C. quinquefasciatus*. However, two genes orthologous to *serrate (ser)*, which also encodes a N ligand, were identified in *C. quinquefasciatus*, while single *ser* orthologs were identified in the other two mosquito species. Components downstream of the receptor were fairly well conserved in mosquitoes. For example, single *hairless* and *suppressor of hairless* genes, which function as transducers in the signaling cascade, were identified in all three mosquitoes. Targets of the pathway, including singular *Enhancer of split E(spl)* genes (but not an *E(spl)* complex like that of *Drosophila*), were identified in the three mosquito species. Functional analysis of the putatative *C. quinquefasciatus N* genes, the two *C. quinquefasciatus ser* orthologs, and these downstream N signaling components in *C. quinquefasciatus* may prove interesting.

#### Janus kinase/signal transducer and activator of transcription (JAK-STAT)

The JAK-STAT pathway has been implicated in a number of *D. melanogaster* developmental processes, such as hematopoeisis, border cell migration during oogenesis, and eye development to name only a few [35]. Components of the JAK-STAT pathway are well conserved in all three vector mosquitoes, with the exception of the rapidly evolving ligand Unpaired, which is not conserved outside of *Drosophila* [25]. Single orthologs of *JAK/hopscotch* were identified in the three mosquitoes. Although a single copy of *Stat92E*, which encodes a transcription factor, was found in *A. aegypti*, three were found in *C. quinquefasciatus*, and two were found in *A. gambiae*. *STAT* duplications were also noted in *A. pisum* [25]. However, while *A. pisum* possesses five *domeless* (*dome*) receptor paralogs [25], the three mosquitoes each have only a single ortholog.

#### Hedgehog (hh)

In flies, Hh signaling regulates a variety of developmental processes, from segmentation and nervous system development to eye and wing development, to name only a few [35]. Components of the Hh signaling pathway are very well conserved in all three mosquito species. Orthologs of genes encoding the ligand Hedgehog, receptor Patched, downstream players Smoothened, Costa, Fused, PKA, the transcription factor Cubitus Interruptus, as well as the repressor Suppressor of fused, are all found in mosquitoes. The only lineage-specific gain noted is that two copies of Slimb, which regulates protein degradation in the ubiquitin-proteasome pathway [36], are found in *C. quinquefasciatus*.

#### Receptor Tyrosine Kinase (RTK)

The three mosquito genomes were examined for components of three RTK signaling pathways, including the Epidermal Growth Factor (EGF), Fibroblast Growth Factor (FGF), and Ras signaling pathways. EGF signaling functions in a variety of developmental processes in *Drosophila*, including the regulation of eye and wing cell differentiation [37], as well as many additional processes [35]. Mosquitoes have orthologs of *spitz* and *keren*, which encode EGF receptor ligands. However, all three mosquito species lack *grk*, which encodes an EGF receptor ligand in flies. Absence of *grk* was also noted in the *A. pisum* and *A. mellifera* genomes, and this gene was not found in the *Tribolium* or *Bombyx* genomes [24], [25]. *argos* was not identified in any of the mosquito genomes. Absence of *argos*, which encodes a negative regulator of EGF signaling [38], is unusual in insects. *D. melanogaster*, *T. castaneum*, and *A. pisum* have single orthologs, while the *A. pisum* genome contains four *argos* genes [24], [25]. Finally, a lineage specific absence of the gene *star*, which functions in EGF processing [39], was also noted in *A. gambiae*.

**Figure 3:**
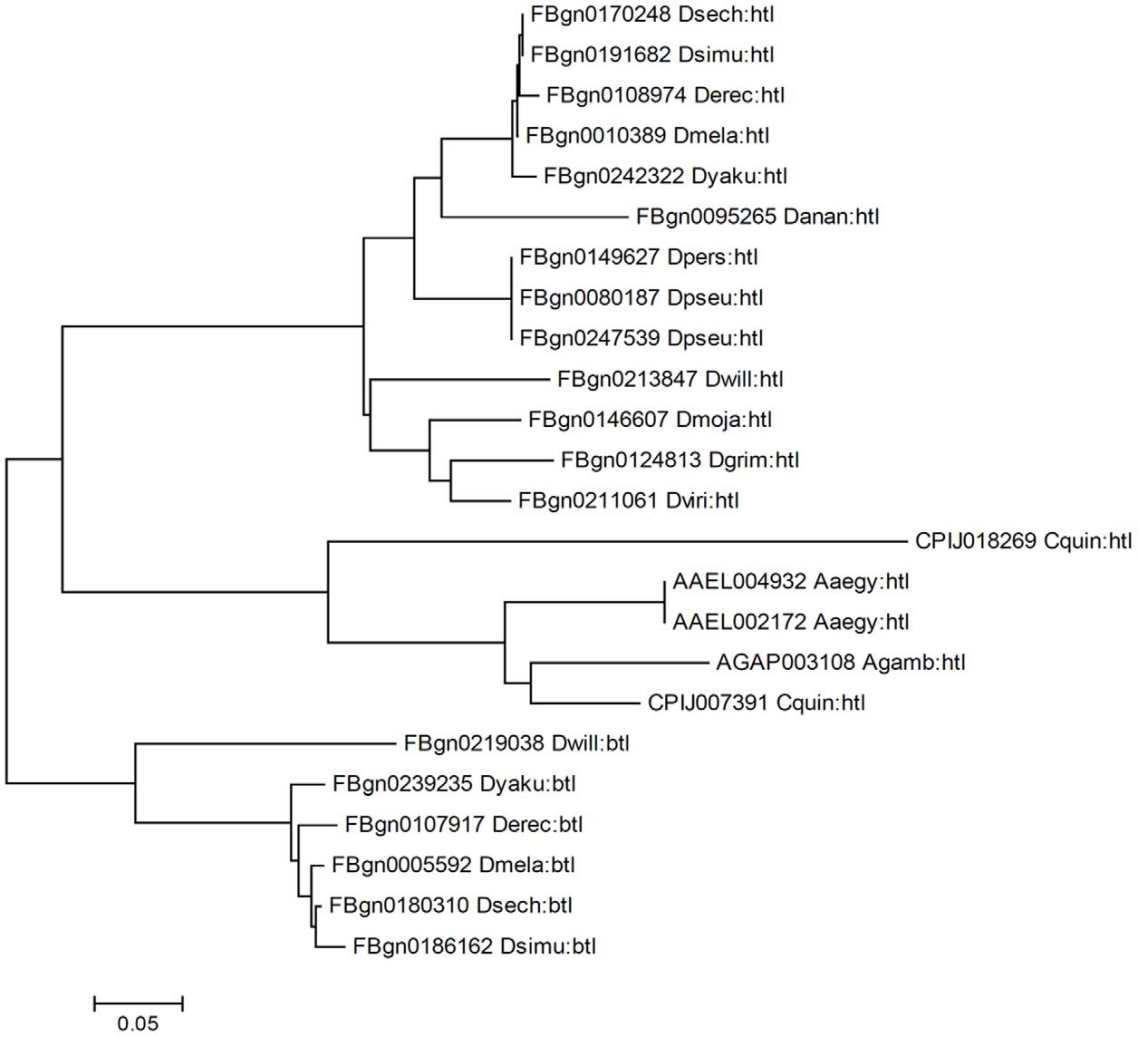
Phylogenetic relationships of FGF receptor genes. *D. melanogaster* has two FGF receptors, Htl and Btl. A Neighbor-Joining tree of Htl and Btl proteins among mosquito and *Drosophila* species is shown. The gene ID and the species name (5 letters) are indicated for the orthologs. The optimal tree (the sum of branch length = 2.022) is shown. The percentage values of replicate trees in which the associated taxa clustered together in the bootstrap test (1000 replicates) are shown next to the branches. The tree is drawn to scale (shown below the tree), with branch lengths in the same units as those of the evolutionary distances used to infer the phylogeny. The distance scale is in units of the number of amino acid substitutions per site. The results of these analyses indicate that mosquito FGF receptors are *htl* orthologs, and mosquitoes lack *btl*.

FGF signaling regulates a variety of biological processes, including cell differentiation and migration, in flies as well as in vertebrates [40]. Several interesting observations resulted from analysis of mosquito FGF signaling pathway components. A single ortholog of the FGF ligand *branchless (bnl)* was identified in *A. gambiae*, and two copies of this gene were found in *C. quinquefasciatus*. However, *bnl* was not identified in *A. aegypti*. Furthermore, *thisbe* and *pyramus*, which function as FGF ligands during *D. melanogaster* development [41], were not identified in *A. aegypti*. *D. melanogaster* has two FGF receptors, *breathless (btl)* and *heartless (htl)*. Mosquito FGF receptors are *htl* orthologs (Figure 3), and mosquitoes lack *btl*. Both *A. aegypti* and *C. quinquefasciatus* have two *htl* genes.

Ras/MapK signaling functions to regulate many developmental processes [35], perhaps most notably fly eye development, in which this pathway has been intensely studied [42]. Components of the Ras/MapK signaling pathway are very well conserved in mosquitoes. Several lineage-specific gains were noted, including: two copies of the MapK-encoding *rolled* and *nemo* genes in *A. aegypti* and two MapKK-encoding *hemipterous* orthologs in *C. quinquefasciatus*. The absence of key Ras pathway components was not noted in any of the mosquito species.

#### Transforming growth factor beta (TGFβ)

In flies, TGFβ signaling has been implicated in many developmental processes, from dorso-ventral patterning in the embryo, where its function was initially described (reviewed in [43]), to the regulation of organ size [44]. Components of the *TGFβ* signaling cascade are generally well conserved in mosquitoes, with a few notable exceptions. Single orthologs of the ligand-encoding gene *decapentaplegic* were identified in each species, though *activin-beta*, which also encodes a ligand, was not identified in *A. aegypti* or *A. gambiae*. Furthermore, *screw*, another member of the *TGFβ* superfamily, was not found in any of the mosquitoes. Van der Zee et al. [45] suggested that *scw* probably arose by duplication of another *TGFβ* superfamily member, *glass bottom boat (gbb)*, between the Culicomorpha and the higher Diptera and underwent rapid divergence. However, *A. aegypti* and *A. gambiae* possess single orthologs of *gbb*, while *C. quinquefasciatus* has two. TGFβ receptors were also analyzed. At least one copy of the receptor-encoding genes *baboon (babo)*, *thickveins (tkv)*, *punt*, and *saxophone (sax)*, were present in all of the mosquitoes, while three genes orthologous to *tkv* and/or *sax* were identified in *A. aegypti*. The SMAD family genes *medea*, *mad*, and *Smox*, which encode transcription factors, were all found in mosquitoes. However, *Dad*, which is present in a number of insect species [25] and encodes an anti-SMAD, was not identified in mosquitoes.

### Genes that regulate fundamental developmental processes

A number of fundamental developmental processes have been studied in *Drosophila*, as well as other animal models for development. Here, genes that regulate the processes of axis formation, segmentation, segmental patterning (Hox genes), germline specification, neurogenesis, and apoptosis were examined. Discussion of these processes centers around the *D. melanogaster* genes that regulate them and comparative analysis of these genes in mosquitoes. Orthology assignments for the genes discussed below are provided in Table S1. Information regarding mosquito lineage specific absences or gains of genes (with respect to *D. melanogaster*) regulating these fundamental processes is summarized below and provided in Table 2. It should be noted here and in subsequent sections of the Results/Discussion that although a particular gene is often discussed in relation to a specific developmental process, many of these developmental genes are pleiotropic and function in a variety of developmental processes. Discussion of a gene in any given developmental context does not mean that its role is limited to a single developing tissue or process, but permits thematic organization of the results, which is necessary given the breadth of this investigation.

**Table 2:** Comparative analysis of genes that regulate fundamental developmental processes.

Process and Gene	*Aae*	*Cqu*	*Aga*	Process and Gene	*Aae*	*Cqu*	*Aga*
**Axis formation**				**Axon Guidance**			
*bcd*	0	0	0	*Abi*	1	2	1
*exu*	1	1	2	*Actbeta*	0	0	1
*grk*	0	0	0	*Ama*	2	1	1
*mago*	2	1	1	*argos*	0	0	0
*swa*	0	0	0	*btn*	0	1	1
*tor*	2	2	1	*CadN*	1	1	2
*Tslr*	0	0	0	*Cam*	1	3	2
				*cas*	1	1	0
**Segmentation**				*Cdk5*	2	1	1
*cnc*	2	1	1	*chb*	1	2	1
*ems*	2	1	2	*comm*	0	0	0
*ftz*	0	1	1	*Con*	2	2	2
*gsb*	0	1	1	*CSN5*	2	1	1
*gsb-n*	0	1	1	*Dab*	2	1	1
*hairy/h*	2	1	1	*eg*	2	2	1
*odd*	1	1	2	*emc*	1	1	2
*pan*	2	3	2	*fas*	1	1	2
*prd*	0	0	0	*fas3*	1	1	2
*wg*	2	1	1	*futsch*	1	2	1
				*gcm*	2	2	2
**Segmental patterning (Hox genes)**				*gl*	0	1	1
*Antp*	1	2	1	*glec*	0	0	0
*HoxR*	0	0	0	*Hem*	2	1	1
*Ubx*	0	1	0	*jing*	1	1	0
*zen*	0	0	0	*Lim1*	0	1	1
				*Mical*	3	3	1
**Germline specification**				*mmy*	1	2	1
*aub*	7	7	2	*N*	1	0	1
*piwi*	0	0	0	*NetA*	3	2	1
*wun*	1	2	1	*NetB*	1	2	1
				*nvy*	2	0	1
**Apoptosis**				*Oda*	1	0	1
*chm*	1	1	2	*Pak*	1	0	1
*Cyt-c-d*	1	2	1	*pasha*	1	2	1
*eff*	1	3	1	*pdm3*	1	2	1
*Eip93F*	2	1	1	*plexB*	2	0	1
*grim*	0	0	0	*Ptp69D*	1	1	2
*hid/W*	0	0	0	*repo*	0	1	1
*lok*	1	1	2	*rho*	0	0	0
*p53*	3	5	2	*robo*	2	2	2
*PSR*	2	1	1	*rst*	2	2	2
*rpr*	0	0	0	*S*	1	1	0
*th*	1	1	5	*Sema-2a*	2	1	2
*wgn*	0	1	1	*shot*	3	4	2
*yki*	3	2	1	*Src42A*	1	0	1
				*Tl*	5	1	7
**Neurogenesis**				*trh*	2	2	1
*N*	1	0	1	*trio*	2	2	1
*Dl*	1	0	1	*tsr*	1	2	1
*cas*	1	1	0	*vvl*	1	2	1
*ac*	0	0	0	*wnd*	1	0	1
*sc*	3	2	1				

The number of orthologous sequences for *D. melanogaster* genes that regulate the processes of axis formation, segmentation, segmental patterning (Hox genes), germline specification, neurogenesis, and apoptosis are indicated for each of the three mosquito species. Although genes regulating these fundamental developmental processes are generally very well conserved, changes in the number of orthologous sequences for several genes implicated in these processes were observed in mosquitoes. Results are reported only for cases in which the number of orthologous sequences varies between *D. melanogaster* and at least one of the mosquito species. Reported numbers refer to the number of orthologous sequences present in the three mosquito genomes for each *D. melanogaster* gene indicated at left.

#### Axis formation

In *D. melanogaster*, terminal patterning and anterior-posterior axis specification is initiated by maternal products localized to the anterior and posterior poles of the embryo. During fly development, binding of Trunk ligand to the receptor tyrosine kinase Torso (Tor) activates a Ras-MapK signaling cascade that represses expression of *tailless* and *huckebein* at the embryonic poles. A homologous pathway functions in early *T. castaneum* development [46]. Multiple *Tor* genes exist in the mosquito genomes, and other key components of the Tor signaling pathway are conserved in all three mosquito species, suggesting that it may regulate terminal patterning in mosquitoes. However, both *A. mellifera* [24] and *A. pisum* lack components of this pathway, indicating that this mechanism of terminal patterning is an evolutionarily derived trait [25].

*Drosophila* dorso-ventral patterning is initiated during oogenesis by Gurken (Grk), an EGFR ligand (Roth, 2003). As discussed in the Receptor Tyrosine Kinase signaling section above, Grk, proposed to be an invention of Diptera [24], is not found in any of the mosquito genomes. However, key downstream components of the dorso-ventral patterning pathway, such as *EGFR*, *pipe*, *Toll*, and *dorsal*, are conserved in mosquitoes. These genes are believed to be part of a dorso-ventral patterning system that is conserved in insects, but which has co-opted *grk* in *Drosophila* [24], [25].

In flies, RNA localization of maternal-effect genes in the oocyte regulates anterior-posterior patterning. At the anterior end of the embryo, *bicoid (bcd)* RNA is localized as a result of the activity of Exuperantia (Exu), Swallow (Swa), and Staufen (Stau). Although mosquitoes possess orthologs of *exu* and *stau*, they lack *bcd* and *swa*. This is not unexpected, as *bcd* is a derived *Hox3* gene found only in higher dipterans [24], [47]. *swa* has not yet been identified in any non-dipteran species [24], and given its absence in mosquitoes, is apparently not found in all diptera. At the posterior end of the *Drosophila* embryo, Oskar, which is thought to be dipteran-specific and is present in all three mosquitoes, functions as an anchor for posterior-determining components [11], [48]. Orthologs of genes whose products function both upstream (*cappuccino*, *mago nashi*, *oo18 RNA-binding protein*, *spire* and *staufen*) and downstream (*nanos*, *par-1*, *pipsqueak*, *pumilio*, *tudor* and *vasa*) of Oskar, all of which are generally well conserved in other insects [24], [25], are found in mosquitoes. *valois*, a gene that is found in many insects, but not in *A. pisum* [25], is present in all three mosquitoes. In summary, although a number of genes that function as anterior-determining components in flies have not been identified in mosquitoes (and other insects), the posterior-determining components are well conserved in mosquitoes.

#### Segmentation genes

Mosquitoes, like fruit flies and other dipterans, are long germ insects. In long germ insects, the germ anlage represents all of the body segments, and these segments are specified simultaneously in the blastoderm. In *D. melanogaster*, a hierarchy of segmentation genes regulates the specification of segments. Maternally-derived mRNAs function at the top of a hierarchy in which the zygotic gap, pair-rule, and segment polarity genes are sequentially activated [23]. Despite the shared mode of segmentation between mosquitoes and fruit flies, a survey of mosquito orthologs for fly segmentation genes uncovered some striking differences.

The products of maternal mRNAs regulate expression of transcription factor-encoding gap genes. Gap genes were initially identified by their *D. melanogaster* loss-of-function mutant phenotypes in which regions of larval cuticle spanning several segments were found to be deleted [23], [49]. Most of the gap genes are very well conserved in all three mosquitoes. However, no *empty spiracles (ems)* gene was identified in *C. quinquefasciatus*. Ems is required for head development [50], [51]. Also, *A. aegypti* possesses two orthologs of *capncollar* (*cnc*), a second head gap gene. Both *ems* and *cnc* are discussed in more detail in the context of head development, a process that has diverged in insects (see below).

The transcription factor-encoding pair-rule genes represent the first periodic gene expression in *D. melanogaster* embryos. The pair-rule genes were identified by their loss-of-function phenotypes which are characterized by cuticular deletions that occur in a two-segment periodicity. The corresponding striped expression of these genes, which also occurs in a two-segment periodicity in both the syncitial and cellular blastoderm, is established by action of the maternal coordinate and gap genes [23], [49]. Several interesting changes in pair-rule gene number were observed in mosquitoes. First, additional copies of several pair-rule genes were noted. These include: two *odd-skipped* genes in *A. gambiae* and two copies of *hairy* in *A. aegypti*. The absences of pair-rule genes were also noted. First, no *paired (prd)* gene ortholog was found in the three mosquitoes, which will be discussed further in relation to other *Pax3/7* segmentation genes (see below). Secondly, no *fushi tarazu (ftz)* gene was identified in *A. aegypti*. Rapid sequence evolution of *ftz* in insects has been noted [24], [25]. It is therefore possible that *A. aegypti* possesses a highly-divergent *ftz* gene that has yet to be identified.

Pair-rule genes regulate the expression of segment polarity genes, which are typically expressed in a segmentally reiterated pattern just following the onset of gastrulation and throughout the morphologically segmented germ band stage. Mutation of these genes results in patterning defects that can be observed in every segment of the cuticle. Segment polarity genes encode a variety of cellular proteins, including transcription factors, as well as ligands, receptors, and other components of signaling pathways, including members of the Hh and Wnt signal transduction cascades (reviewed in [23]). The segment polarity genes are generally very well conserved in mosquitoes (see also Wnt and Hh signaling discussion above) with one exception, absence of the Pax3/7 gene *gooseberry* in *A. aegypti*.

*D. melanogaster* possess three *Pax3/7* genes: the pair-rule gene *prd*, the segment polarity gene *gsb*, and *gooseberry-neuro (gsb-n)*, a gene that is expressed in the embryonic CNS [52]. The number of *Pax3/7* orthologs is known to vary among arthropods. For example, the grasshopper *Schistocerca americana* has two *Pax3/7* genes, *pairberry1 and 2 (Sa-pb1 and Sa-pb2)*. *Sa-pb1* is transiently expressed in a pair-rule fashion before resolving into a segmental pattern coincident with its paralog, *Sa-pby2* [53]. Therefore, the two *Pax3/7* genes identified in both *C. quinquefasciatus* and *A. gambiae* may similarly serve both pair-rule and segment polarity gene functions. However, the inability to identify any *Pax3/7* orthologs in *A. aegypti* is highly unusual, as we are unaware of other insects that lack a *Pax3/7* gene.

#### Hox complex genes

The Hox complex genes specify segment identity along the anterior-posterior axis during metazoan development [35]. Hox cluster genes were examined in the three mosquito species. Two copies of *Antp* were identified in *C. quinquefasciatus*. A single copy of *Ubx* was identified in *C. quinquefasciatus*, but *Ubx* was not identified in *A. gambiae* or *A. aegypti*. As mentioned above, *A. aegypti* lacks *ftz*, and orthologs for *zerknüllt* (*zen*) were not found in the three mosquito species. Both *zen* and *ftz* have evolved non-homeotic functions in insects, and in *A. pisum*, the amino acid sequences from these genes were too divergent to permit unambiguous orthology assignments through phylogenetic analysis. Instead, orthology assignments for *A. pisum ftz* and *zen* rested principally on their genomic locations next to *Scr* and *pb*, respectively [25]. For *ftz*, no open reading frame was identified in a comparable position in *A. aegypti*. However, a *zen2* ortholog was found to be located next to *pb* in *C. quinquefasciatus* and *A. gambiae*; an ortholog of this gene was identified in *A. aegypti*, though it does not appear to be located within the Hox complex in this species.

#### Germline specification

Germline cells are separated from somatic cells during early embryogenesis of many different species. In flies, germline cells form through incorporation of pole plasm, which is assembled in the posterior pole of the oocyte during oogenesis. The pole plasm contains a number of RNAs and maternal proteins that function to specify germline cell fate in the early *D. melanogaster* embryo through regulation of processes such as translation and mRNA localization. In *Drosophila*, germline specification, a process that is well-conserved across many different species, yields 20–30 primordial germ cells (PGCs; also referred to as pole cells in *Drosophila*) (reviewed by [54]). Nanos, Oskar, Vasa, and Tudor, all of which are key players during fly germline development, are present in vector mosquitoes, where they may play conserved roles during germline development.

*A. aegypti* and *Culex* have undergone expansion of the Argonaute/PIWI subfamily genes. These findings were reported in a previous study [55], which concluded that the *A. aegypti* and *Culex* genes are evolving faster than those of *A. gambiae* and *D. melanogaster* [55]. Given their developmental importance, these genes are included as part of our cumulative data set (Table S1), and the roles of these genes during germline development are briefly reviewed. Piwi and Aubergine (Aub) are essential for germline stem cell maintenance in adult *Drosophila* ovaries and testes. In the germline, these proteins associate with 24–32 nucleotide small RNAs known as PIWI-interacting RNAs (piRNAs) which function in gene silencing. PIWI proteins are critical during germline development and gametogenesis in many metazoan species, including germline determination and GSC maintenance, meiosis, spermiogenesis, and transposon silencing (reviewed by [56]. Given the rapid evolution of these proteins, it will be interesting to functionally assess their roles in mosquitoes.

#### Neurogenesis

Genes of the *Drosophila achaete-scute (ac-sc)* cluster, which include *achaete (ac)*, *scute (sc)*, *lethal of scute [l(1)sc]*, and *asense (ase)*, encode basic helix-loop-helix (bHLH) transcription factors that induce neuronal fate [57]. An *ac* ortholog was not found in any of the mosquitoes, which do however possess a number of other *ac-sc* cluster proneural gene orthologs: four in *A. aegypti*, two in *C. quinquefasciatus*, and two in *A. gambiae* (see Table S1 for ortholog assignments). Unlike flies, these genes are not clustered together in either *A. aegypti* or *C. quinquefasciatus*, though the two *A. gambiae* genes are located next to each other. Although the number of genes varies from species to species, studies in *Drosophila* have demonstrated that a high degree of functional redundancy of the products of the *ac-sc* cluster exists, and that the bHLH domain is necessary and sufficient to mediate the proneural function, activate neurogenic genes, and allow lateral inhibition [58].

Axon guidance genes are generally well conserved in mosquitoes. For example, *frazzled (fra)*, *Down syndrome cell adhesion molecule*, *slit*, *roundabout (robo)*, *robo3*, *ephrin*, *longitudinals lacking*, *semaphorin*, and *plexin* genes, all of which function during fly nervous system development (reviewed in [35], [59]), are found in all three mosquito species. However, a *commissureless (comm)* ortholog was not found in any of the mosquitoes. *A. aegypti* does have a gene that resembles *comm2* and *comm3*, and *C. quinquefasciatus* has a total of three genes resembling both *comm2* and *comm3*. However, neither *comm2* nor *comm3* genes were found in *A. gambiae* [26], an interesting observation given that *comm* is critical for embryonic ventral nerve cord development in *Drosophila* (reviewed in [59]). Absence of *comm* in the *A. gambiae* lineage suggests that mechanisms for generating a nerve cord may have diverged between fruit flies and mosquitoes, a hypothesis that is supported by our recent work ([18]; Haugen et al., submitted).

The *A. aegypti* and *C. quinquefasciatus* genomes contain multiple copies of the axon guidance gene *netrin (net)*. In contrast, only one copy of both *netA* and *netB* are present in *D. melanogaster* and *A. gambiae* (Figure S1). In *Drosophila*, both *netA* and *netB* are expressed by midline cells in a largely overlapping pattern of expression and function to regulate commissural axon guidance at the fruit fly midline [60], [61]. Although a cross-reactive antibody detected Net expression at the midline of *A. aegypti* in a pattern roughly comparable to that of *Drosophila* and other arthropods [16], detailed expression analysis of individual *Aae net* genes in the nervous system or other developing tissues has not yet been investigated in this species. However, siRNA-mediated functional analysis of the Net receptor *fra* suggests that although Fra plays a critical role during development of the *A. aegypti* ventral nerve cord, the *A. aegypti* knockdown phenotype is stronger than that of the *D. melanogaster fra* null mutant. These observations suggest that regulation of embryonic commissural axon guidance might differ in the two insects [18]. It is therefore possible that lineage specific amplification of *net* genes in Culicine species may have contributed to these differences.

A number of other lineage-specific axon guidance gene absences were noted. *p21-activated kinase (Pak)* and *nervy (nvy* ) were not identified in *C. quinquefasciatus*. In flies, Pak localizes to axons and growth cones and functions as a critical regulator of axon guidance [62]. Nvy couples cAMP-PKA signaling to PlexA to regulate Sema-1a-mediated axonal repulsion, thereby allowing growing axons to integrate inputs from multiple guidance cues [63] in *Drosophila*. Furthermore, *scribbler (sbb)*, which is required for axonal guidance in the *Drosophila* visual system [64], [65], was not found in *A. aegypti* or *A. gambiae*. Finally, *jing*, a gene that is required for proper CNS development in flies [66], was not identified in *A. gambiae*.

#### Apoptosis

**Figure 4:**
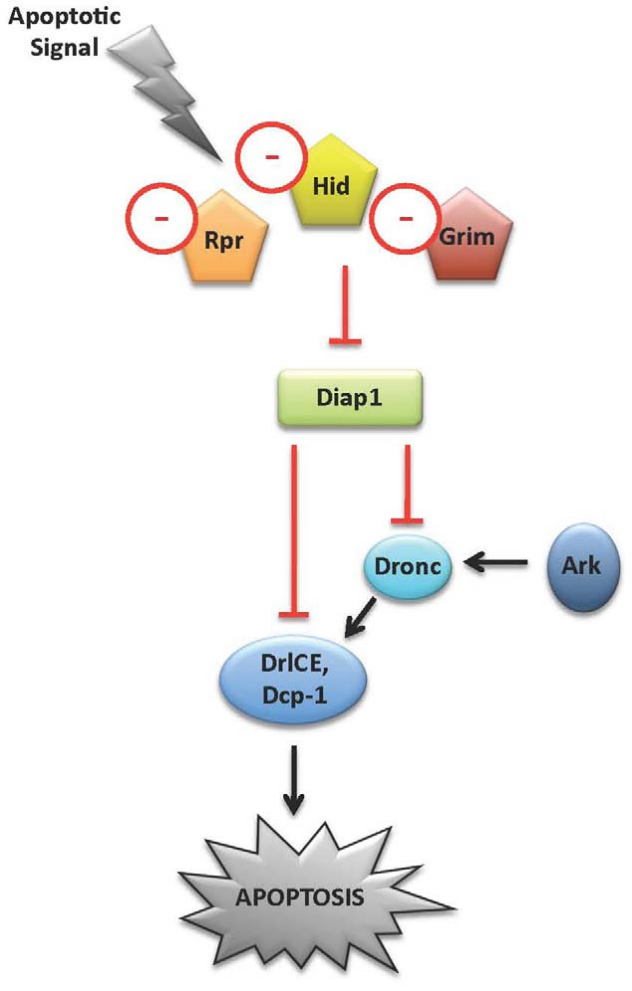
Regulation of cell death in dipterans. In *D. melanogaster*, apoptosis is induced by the proapoptotic proteins Grim, Rpr, and Hid, which antagonize the function of dIap, thereby preventing it from inhibiting Dronc. When Dronc is active, DrICE, the main effector caspase of apoptosis in flies, is activated (reviewed in [67], [68]). Although many key components of the *Drosophila* apoptosis pathway are conserved in mosquitoes, mosquitoes lack orthologs of several key regulators of apoptosis (denoted by a – sign). In mosquitoes, Michelob_X is believed to function as the missing Iap antagonist [73]. Additional details and discussion are provided in the text.

In *D. melanogaster*, apoptosis is induced by three proapoptotic proteins, Grim, Reaper (Rpr), and Head involution defective (Hid), which function as Inhibitor of apoptosis protein (Iap) antagonists. Such antagonists prevent Iap from inhibiting Dronc. Activated Dronc, which requires activity of the adaptor protein Ark, cleaves and activates DrICE, the main effector caspase of apoptosis in flies (Figure 4; reviewed in [67], [68]). Bryant et al. [69] annotated apoptosis-related genes in *A. aegypti* and *A. gambiae*. Given the importance of programmed cell death in relation to development, their ortholog assignments are included here, and relevant ortholog assignments from *C. quinquefasciatus* have been added. In general, the core pathway regulating apoptosis in *A. aegypti* bears many similarities to that of *D. melanogaster*. Orthologs for many key pathway components, such as *Iap1*, *Iap2*, *Dronc*, *Dredd*, *Ark*, and *Ice*, exist in *A. aegypti* [69], [70], [71], [72], *C. quinquefasciatus*, and *A. gambiae*. However, orthologs for *grim*, *rpr*, and *hid* (results are summarized in Figure 4), which have not yet been found outside of *Drosophila* species, are absent in mosquitoes. Zhou et al. [73] suggest that Michelob_x (which is not found in *Drosophila*) plays an equivalent role in mosquitoes. Liu and Clem [74] also suggest that the effector caspase Dcp1, which functions in a tissue-specific manner in flies [75], plays a more significant role in *A. aegypti*. *Dcp1* orthologs are also present in *C. quinquefasciatus* and *A. gambiae*.

### Genes regulating development of tissues of vector importance

While detailed and thorough investigation of all aspects of mosquito development is critical, analysis of tissues that are vital to host location and the spread of infection is of global health importance. Blood-feeding mosquitoes rely on their olfactory systems for host location. Disease causing viruses and parasites are ingested in the blood meal and replicate in the midgut epithelium (reviewed by [76]). Despite natural anatomical barriers for pathogen dissemination, including cuticle proteins and cuticle-domain proteins [77], [78], infection can spread to secondary sites in the mosquito body and eventually the salivary glands. Following infection of the salivary gland, female mosquitoes remain competent for disease transmission for the duration of their lives (reviewed by [76]). The genetics of *Drosophila* salivary gland, olfactory system, and cuticle development is summarized below. Relevant mosquito ortholog assignments for *Drosophila* genes of interest are provided in Table S1, and information concerning mosquito lineage-specific absences and gains of genes (with respect to *D. melanogaster*) regulating the development of these tissues in flies is provided in Table 3.

**Table 3:** Analysis of genes that regulate the development of tissues of vector importance.

Tissue and Gene	*Aae*	*Cqu*	*Aga*
**Salivary Gland**			
*a*	1	2	1
*bib*	1	1	2
*Cam*	1	3	2
*Hr46*	2	1	1
*egl*	1	2	1
*fkh*	2	1	1
*klar*	1	2	2
*sens*	1	2	1
*odd*	1	1	2
*scb*	2	1	2
*shg*	2	1	3
*Btk29A*	2	1	1
*trh*	2	2	1
*ash1*	1	2	1
*htl*	2	2	1
*Eip63E*	2	1	1
*esg*	0	0	0
*eyg*	0	1	1
*Wnt4*	2	1	1
*Awh*	4	4	3
*Chi*	2	1	1
*Eip93F*	2	1	1
*jumu*	1	1	2
*JIL-1*	1	2	1
*brk*	0	1	1
*Smr*	0	1	1
*par-6*	1	2	1
*pvf2*	0	0	1
*mod*	0	0	0
*src42A*	1	0	1
*N*	1	0	1
*Pvf3*	1	1	0
**Olfactory System**			
*ac*	0	0	0
*ato*	3	3	2
*CadN*	1	1	2
*dac*	1	3	1
*N*	1	0	1
*robo*	2	2	2
*sc*	3	2	1
*toy*	1	2	1
*wg*	2	1	1
**Larval Cuticle**			
*Ccp84Ad*	15	2	1
*CG7203*	10	4	3
*Cpr30B*	6	1	1
*Cpr30F*	9	2	3
*Cpr65Eb*	9	6	5
*Cpr76Bb*	2	0	1
*Lcp65Ac*	9	6	4
*Pcp*	1	1	0

The number of mosquito orthologs of genes related to salivary gland and olfactory system development, as well as cuticle components are indicated for the three mosquito species examined in this study. Numbers refer to the number of orthologous sequences present in the three mosquito genomes for each *D. melanogaster* gene indicated at left. Results are reported only for cases in which the number of orthologous sequences varies between *D. melanogaster* and at least one of the mosquito species. Although the genes are generally well conserved, changes in the number of orthologous sequences for several *D. melanogaster* genes implicated in these processes were observed in mosquitoes.

#### Salivary gland

Salivary gland proteins are major components of mouth anatomy that undergo selective pressures among different insects to adapt to specific feeding behavior and host types [79], [80], [81]. Unlike many other hematophagous arthropods, mosquito salivary glands secrete enzymes that aid in sugar feeding [82] and antimicrobial agents to control bacterial growth in the sugar meal [83]. Convergent evolution is believed to play a major selection force in lineage specific adaptation of salivary glands in mosquitoes [84]. Such lineage specific adaptation is manifested in the genes that are functional in salivary glands among species. In *D. melanogaster*, salivary secretory genes are major components of genes that produce secretory proteins present in the saliva of fruit flies but that are absent in mosquitoes [85]. In contrast, the D7 proteins that are ubiquitous in mosquito salivary glands [84] are absent in *Drosophila*. Given these differences in the adult salivary glands of fruit flies and mosquitoes, it is predicted that changes in salivary gland development will also be observed.

The *Drosophila* salivary gland has emerged as an excellent model system for studying the genetics of cell fate specification, cell shape changes associated with tube formation and elongation, and the coordinated migration of an organized developing tissue to its final position within the organism [85]. In contrast, the developmental genetics of mosquito salivary gland development has yet to be investigated. Most genes known to function during development of *D. melanogaster* salivary gland development [35] have orthologs in vector mosquitoes. However, several genes that function during development of the fly salivary gland were not found in mosquitoes. For example, *escargot (esc)*, *modulo (mod)*, and *zeste (z)*, are absent in all three mosquito genomes. Although these genes have been implicated in *Drosophila* salivary gland development, they have not yet been identified in non-*Drosophila* arthropod species. In the *D. melanogaster* salivary gland, overexpression of the transcriptional regulator *esc* inhibits endoreplication, the replication of DNA in the absence of cell division that produces polytene chromosomes, suggesting that it may regulate this process [86]. *mod* is expressed in the secretory cells during fly salivary gland development (reviewed by [87]), but its function there has not yet been assessed. Finally, *z*, a transcriptional regulator, has high levels of expression in the late third instar and pupal salivary gland, and it has been localized to polytene chromosomes [88].

Several mosquito lineage specific gene absences were also noted. *brinker (brk)* and *eyegone (eyg)*, which function during development of the fly salivary gland, were not found in *A. aegypti*. In flies, mutations in *brinker* result in reduction of the salivary gland placode along both the anterior-posterior and dorso-ventral axis, suggesting that this gene functions to pattern both of these axes during salivary gland development [89]. In *Drosophila eyg* mutant embryos, the duct primordia fail to converge and extend across the midline, which results in the absence of individual ducts. Many individual presumptive duct cells join with the presumptive common duct cells to form an unusually large common duct that fails to connect to the glands in these mutant embryos [90]. Furthermore, the *Niemann-Pick Type C-2a* gene, which is expressed during fly embryonic salivary gland development, was not found in *A. gambiae*. A number of gene gains with respect to *D. melanogaster* were also observed in mosquitoes. For example, four copies of the *arrowhead (awh)* gene were observed in both culicine mosquitoes, while *A. gambiae* has three copies. In flies, which have a single copy of the gene, Awh is required for the generation of histoblast nests, precursors of certain abdominal structures, including the salivary gland [91].

#### Olfactory system

The insect olfactory pathway, in which olfactory neurons located in the maxillary palps and antennae project to distinct glomeruli in the primary olfactory center, shares the general layout of the vertebrate olfactory system. However, as a result of significant reduction in the number of odorant receptor neurons, odorant receptors, and antennal lobe glomeruli, insects, and with respect to genetics *Drosophila* in particular, are exceptionally well-suited for studying the principles of olfactory wiring. Furthermore, the developing larval olfactory system is an increasingly popular system for olfactory analyses, as it shares the design and types of neurons of its adult counterpart, but is even more simplified in terms of cell number (reviewed by [92]).

A number of genes that regulate wiring of the olfactory system have been identified in flies [35]. Many of these genes, including axon guidance genes such as *sema1a* and *lola* that have been implicated in olfactory development [93], [94], have orthologs in all three mosquitoes. In several cases, extra copies of genes known to regulate olfactory development in flies are found in various mosquito lineages. For example, three copies of *dachshund*, a gene that is expressed by olfactory neural precursors as they undergo terminal differentiation in flies [95], are found in *C. quinquefasciatus*. *absent MD neurons and olfactory sensilla (amos)* was not found in any of the mosquito lineages. In flies, *amos* is a proneural gene required to establish the identity of the solo-MD neurons and to establish the identity of two olfactory sensilla: basiconica and trichodea sensilla. *amos* is a proneural gene for a subset of olfactory sensilla, most likely the sensilla basiconica and trichodea [96]. *lim-1*, a gene that regulates dendritic targeting of projection neurons [97] is absent in *A. aegypti*. A number of genes that function to regulate antennal development in flies were also absent in mosquitoes. *distal antenna-related*, a transcriptional regulator that controls differentiation of distal antennal structures [98], was not found in the mosquito species. Finally, *pleiohomeotic*, a gene that regulates expression of genes during antennal disc development [99], was not identified in *A. aegypti* or *A. gambiae*.

#### Cuticle

**Figure 5:**
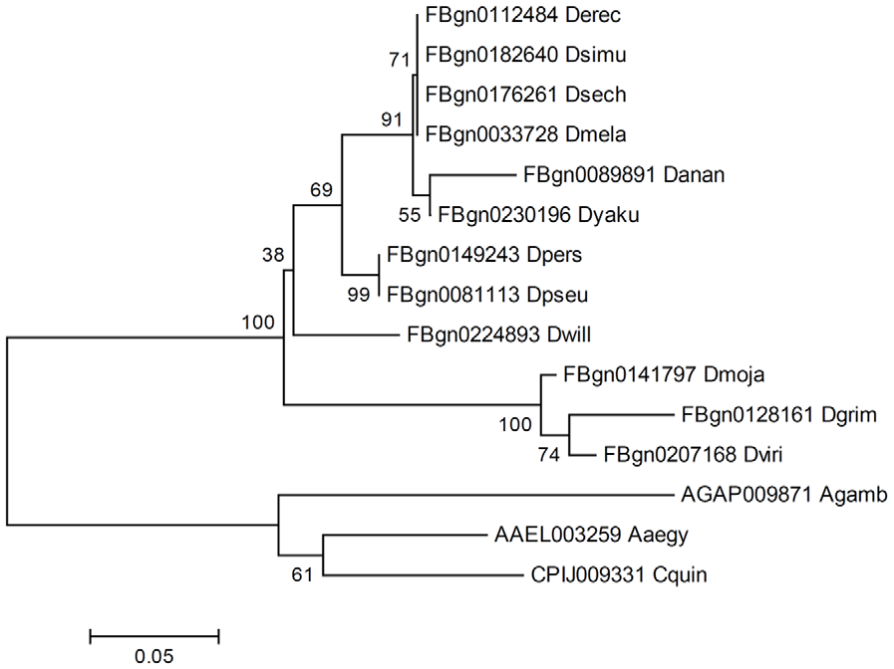
Cuticle Gene Phylogenetic Relationships. Evolutionary relationships of adult cuticle protein genes (one-to-one orthologs) among mosquito and *Drosophila* species were inferred using the Neighbor-Joining method. The gene IDs and the species names (5 letters) are shown for the orthologs. The optimal tree (the sum of branch length = 0.842) is shown. The percentage values of replicate trees in which the associated taxa clustered together in bootstrap testing (1000 replicates) are shown next to the branches. The tree is drawn to scale (shown below the tree), with branch lengths in the same units as those of the evolutionary distances used to infer the phylogeny. The distance scale is in units of the number of amino acid substitutions per site. Although fruit fly and mosquito cuticle proteins are a highly conserved family of proteins, the one-to-one orthologs show lineage specific phylogenetic groupings among the 12 fruit fly and three mosquito species.

The roles of cuticle proteins and cuticle-domain proteins in response to microbial challenge has been described in *A. gambiae* [77]. It is hypothesized that they contribute to the anatomical barriers for pathogen dissemination in a fashion comparable to the dengue virus midgut escape barrier of *A. aegypti* [78]. The tracheal system makes intimate contacts with midgut epithelial cells that act as a dissemination conduit for insect/virus interaction [100]. Trachea contain a cuticular lining that limits virus dissemination [101], and induction of cuticle proteins during development may inhibit the pathogen dissemination process in mosquitoes. Although cuticle proteins are highly conserved families of proteins among fruit flies and mosquitoes, the one-to-one ortholog genes among the 12 fruit fly and three mosquito species show lineage specific phylogenetic groupings in the tree (Figure 5).

### Genes that function in developmental processes that have diverged within insects

Comparative developmental studies have uncovered a number of divergent developmental processes in insects. Here, four such processes are considered: sex determination, dosage compensation, head development, and egg diapause. Orthology assignments for the genes discussed below are provided in Table S1. Information regarding mosquito lineage specific absences and gains of genes (with respect to *D. melanogaster*) regulating these processes is summarized below and included in Table 4.

**Table 4:** Comparison of genes that regulate developmental processes which have diverged in insects.

Process and Gene	*Aae*	*Cqu*	*Aga*
**Sex Determination – Dosage Compensation**			
*fl(2)d*	1	2	0
*JIL-1*	1	2	1
*msl-1*	0	1	0
*msl-2*	1	2	1
*Oda*	1	0	1
*sc*	3	2	1
*sisA*	0	0	0
*snf*	2	1	1
*tra*	0	0	0
*tra2*	4	1	1
**Head development**			
*Akap200*	1	0	1
*Antp*	1	2	1
*aPKC*	1	1	3
*argos*	0	0	0
*bcd*	0	0	0
*bib*	1	1	2
*bnl*	0	2	1
*cnc*	2	1	1
*croc*	2	1	1
*D*	1	0	1
*Dl*	1	0	1
*Dll*	2	1	1
*exd*	2	3	1
*eya*	2	2	1
*fkh*	2	1	1
*gsb*	0	1	1
*grim*	0	0	0
*hdc*	2	2	1
*hig*	1	2	1
*inv*	0	0	1
*Itp-r83A*	2	3	1
*Lim1*	0	1	1
*N*	1	0	1
*pan*	2	3	2
*pho*	0	1	0
*Pkc53E*	1	3	1
*Poxn*	0	1	1
*qtc*	2	1	1
*raps*	2	1	1
*rho*	0	0	0
*rpr*	0	0	0
*rst*	2	2	2
*salm*	0	1	1
*sd*	2	1	1
*Ser*	1	2	1
*ss*	1	0	1
*tkv*	2	1	1
*tld*	2	2	1
*to*	1	1	3
*tor*	2	2	1
*W/hid*	0	0	0
*wg*	2	1	1
*Wnt4*	2	1	1
**Egg Diapause**			
*DopR*	1	2	1

The number of mosquito orthologs for particular genes known to regulate sex determination, dosage compensation, head development, and egg diapause are indicated. Numbers refer to the number of orthologous sequences present in the three mosquito genomes for each *D*. melanogaster gene indicated at left, and results are only reported for genes in which the number of orthologous sequences varies between *D*. melanogaster and at least one of the mosquito species.

#### Sex determination

In *D. melanogaster*, the sex chromosome: autosome ratio signals somatic sex determination through regulation of *Sex-lethal* (*Sxl*), which encodes a protein that is active in females in which it regulates splicing of *transformer* (*tra*, [102]). The splice form containing the complete *tra* open reading frame is female specific. Tra, in conjunction with the constitutively expressed protein Tra2, regulates differential splicing of *doublesex (dsx)*. Sex-specific *dsx* transcripts regulate the differentiation of sexually dimorphic traits [103], [104], [105].

*A. aegypti* and other culicine mosquitoes lack heteromorphic sex chromosomes [106]. Instead, sex is controlled by an autosomal locus wherein the male-determining allele, *M*, is dominant. The primary signal at the top of the mosquito sex determination cascade is therefore different from that of *D. melanogaster*, where the sex chromosome: autosome ratio controls sex differentiation. However, conservation of function in mosquito orthologs of *Drosophila* genes functioning downstream of this signal has been predicted, and several have verified the presence of a number of these genes, including the key players such as *sxl* and *dsx*, in vector mosquitoes [3], [107]. However, *tra* a gene thought to be a key upstream component of an ancestral sex-determining pathway [108], was not found in the three mosquito genomes. The mosquitoes all possess at least one ortholog of *tra2*, which encodes a direct partner of Tra in flies, and *A. aegypti* actually has four *tra2* orthologs. It will be interesting to determine if any of the *tra2* orthologs are differentially spliced in mosquitoes. It should be noted that *A. mellifera*, like mosquitoes, also lacks a *tra* gene but has a *tra2* ortholog [24]. It has been suggested that the *A. mellifera complementary sex determiner* gene can functionally replace *tra* [24], [109]. However, mosquitoes do not appear to have orthologs of this gene, which is not surprising given that the mechanisms of sex determination in honey bees and mosquitoes differ [24].

In mosquitoes, like *A. mellifera*, *sisterless A (sis-A)* is missing. In flies, the ratio of the gene products of three X-linked genes, including *sis-A*, is used to assess the sex-determining X: autosome ratio. It is therefore not unexpected that mosquitoes and *A. mellifera*, which do not use this mode of sex-determination, might not possess orthologs of *sis-A*. To date, *sis-A* has not yet been identified outside of *Drosophila* species. *hermaphrodite (her)*, which appears to be specific to *Drosophila*, was not identified in mosquitoes. In flies, the female-specific Dsx protein (Dsx F) acts in conjunction with Her and Intersex to repress male differentiation and to promote female differentiation in females [110].

#### Dosage compensation

In *D. melanogaster*, the sex-determination cascade controls dosage compensation, which is regulated by a twofold increase in X chromosome transcription [111]. Zdobnov *et al.* [26], who first described dosage compensation gene orthologs in the *A. gambiae* genome, concluded that the basic protein machinery of the dosage compensation complex is conserved between *Drosophila* and *Anopheles*, presumably facilitating flexibility in the evolution of the sex chromosome. In flies, *Sxl*, in combination with *female lethal d [fl(2)d]* and *virilizer (vir)*, controls dosage compensation via *male specific lethal-2* (*msl-2*). All three mosquitoes have *Sxl*, *vir, and msl-2* genes, and *fl(2)d* is missing only in *A. gambiae*. Mosquitoes possess several other fly dosage compensation genes, including *maleless*, *males absent on the first*, *male specific lethal-3*, and *Trithorax-like*, all of which are also conserved in *A. mellifera* [24]. However, several other dosage compensation genes are absent in both mosquitoes and *A. mellifera*, including *roX1* and *roX2* in all three mosquitoes, *ornithine decarboxylase antizyme* in *C. quinquefasciatus*, as well as *male specific lethal-1* in *A. aegypti* and *A. gambiae*.

#### Head development

During *D. melanogaster* embryogenesis, the head is internalized into the thorax during a process called head involution. This results in a highly derived and reduced head as compared to other insect species, including mosquitoes (reviewed in [112]). The mosquito genomes were examined for orthologs of genes known to regulate head development in flies. Of these, the pro-apoptotic genes, including *reaper (rpr)* and *head involution defective* (*hid*; see discussion above) are notably absent. Apoptosis plays a critical role during development of the fly head, where domains of high incidence of cell death are marked by expression of proapoptotic genes such as *rpr*. These apoptotic zones correlate with regions involved in formation of mouth structures, the internalization of neural progenitors, and head involution, the areas where most morphogenetic movements occur [113]. In flies, loss of *rpr* function is associated with the failure of head involution [113], whereas loss of *hid* results in a failure of the dorsal folds to migrate to the anterior [114]. *Lim1*, another gene expressed during fly head development, was also not identified in any of the mosquito species. In fly embryos, *Lim1* is expressed in the head primordia, the brain lobes, and ventral nerve cord. *Lim1* mutants are pupal lethal. Morphologically, *Lim1* mutants appear normal, however mutant larvae display coordination defects and do not crawl in a wild-type fashion [115].

As mentioned above, no ortholog of the gap gene *ems* was identified in *C. quinquefasciatus*. Ems, which is required for brain morphogenesis in flies [50], also functions in conjunction with *orthodenticle (otd)* and *buttonhead (btd)* during head formation. These genes are required for development of the antennal sense organs, as well as the dorso-medial and dorso-lateral papillae of the antennomaxillary complex [51]. *Otd* orthologs were identified in each mosquito. Although *Tribolium*, *Nasonia* and *A. mellifera* all have two paralogs of *otd* [116], [117], mosquitoes have only a single copy of this gene. Mosquitoes also posses single copies of *btd*. Btd regulates *cnc* activation, and Cnc regulates genes responsible for labral and mandibular development, more specifically in the dorsal portion of the labral segment and the posterior lateral and ventral portion of the mandibular segment [118]. Interestingly, *A. aegypti* possesses two *cnc* orthologs, and it is therefore possible that one of these genes has taken on novel roles in this species.

#### Egg diapause

Egg diapause, which can be influenced by both photoperiod and temperature, is a critical adaptation to seasonal environmental variation in a wide range of arthropods [119]. In *A. aegypti*, a container-breeder that lays eggs which are subject to dessication, egg diapause increases dessication resistance. This adaptation is also beneficial in the laboratory, as it allows for collection of *A. aegypti* eggs on artificial substrates and their subsequent storage for several months, after which they can be induced to hatch in deoxygenated water [7]. Egg diapause has been observed in a number of other insect species, including the silkworm *Bombyx mori*, where it has been particularly well studied (see below), but is not found in *D. melanogaster*, *C. quinquefasciatus*, or *A. gambiae*. A literature search identified a number of genes that have been implicated in egg diapause, and the mosquito genomes were examined for orthologs of these genes.

Several groups have studied the genetic regulation of egg diapause in *B. mori* and other insects. Circadian genes, which are photoperiod responsive, have been implicated in the regulation of egg diapause [120]. These genes, which are well conserved in flies and the three mosquitoes, may play similar roles in the regulation of *A. aegypti* and *Bombyx* egg diapause. Environmental stimuli such as photoperiod and temperature ultimately regulate Pheromone biosynthesis activating neuropeptide, the *Bombyx* egg diapause hormone, a key regulator of egg diapause in this species. Diapause hormone is released by the subesophageal ganglion (SG) and induces diapause in developing oocytes, which results in embryonic diapause [121]. The gene encoding Diapause hormone is conserved in all three mosquitoes, but no ortholog was found in the fruit fly. Dopamine signaling, a regulator of the egg diapause hormone, has also been implicated in *Bombyx* egg diapause [122]. Components of this signaling pathway, including two dopamine receptors, *DopR* and *DopR2*, were identified in flies and all three mosquitoes. *C. quinquefasciatus* has two copies of *DopR*. It will be interesting to determine if changes in dopamine or egg diapause hormone signaling underlie the divergence of the egg diapause trait observed in insects, or potentially the timing of the induction of embryonic diapause, which varies temporally among insects that undergo egg diapause.

### Sequence Evolution of Developmental Genes

**Figure 6:**
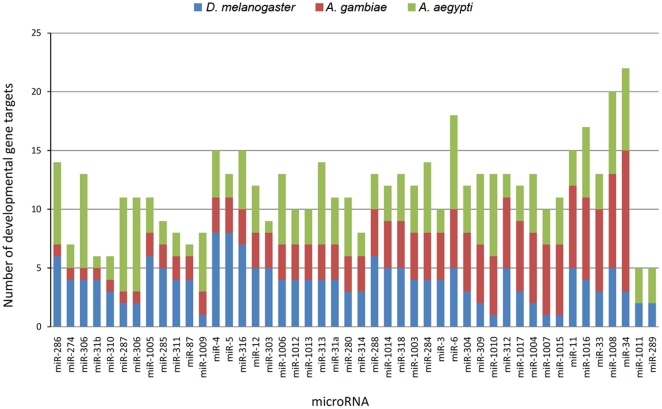
Targets of miRNAs in mosquito developmental genes. The number of predicted developmental gene targets of miRNAs vary in number in *D. melanogaster*, *A. aegypti*, and *A. gambiae*. These results suggest that the repertoire of miRNA developmental gene targets may be species specific.

Several analyses pertaining to coding sequences and untranslated regions (UTRs) of one-to-one orthologous developmental genes between *Drosophila* and mosquitoes were performed. Here, one-to-one orthologs are defined as a single gene representation of homologous genes for the indicated species that may have diverged from a common ancestral gene. Analyses performed included estimates of the coefficients of evolutionary differentiation (Figure S2), evolutionary rates (Figure S3), analysis of repetitive codon sequences (Table 5, Figure S4), and analysis of microRNA (miRNA) binding sites (Figure 6, Figure S5).

**Table 5:** Number of amino acid repeat residues among the one-to-one orthologous developmental genes in the three mosquito species.

Repeats of amino acid	*Aae*	*Aga*	*Cqu*
**Ala**	519	817	504
**Arg**	349	453	332
**Asn**	315	321	255
**Asp**	324	373	285
**Cys**	61	66	53
**Gln**	378	523	343
**Glu**	413	469	380
**Gly**	546	983	569
**His**	173	261	154
**Ile**	253	268	226
**Leu**	732	868	646
**Lys**	356	352	313
**Met**	78	99	78
**Phe**	133	141	125
**Pro**	489	619	423
**Ser**	975	1097	845
**Thr**	379	461	325
**Trp**	15	20	13
**Tyr**	110	122	92
**Val**	323	400	284

The numbers reported correspond to the total count of repetitive residues found in the developmental genes of each species. Mosquito developmental genes contain numerous amino acid repeats, with serine repeats being most common. As discussed in the text, numerous repetitive sequences are a common structural feature of *Drosophila* and mosquito developmental genes.

#### Patterns of evolutionary differentiation

We analyzed molecular evolution of developmental genes that had one-to-one orthologous relationships among the three mosquitoes and the twelve fruit fly species. Estimates of the coefficients of evolutionary differentiation are shown in Figure S2. Based on amino acid substitution patterns, the one-to-one orthologous developmental genes show contrasting evolutionary patterns among the three mosquitoes. The results of these analyses demonstrated that although retaining one-to-one orthology among genomes meant the genes might have crucial functional roles in each of the species, their sequence divergence did not necessarily reflect similar molecular evolution among the species. In future studies, it will be interesting to study the expression profiles of these (and additional) developmental genes during the course of development. Previous studies [123] evaluating the life cycle transcriptome of *A. gambiae* have demonstrated that the coding sequence similarity of orthologues is not correlated with their temporal developmental expression profiles. It is believed that expression profiles and coding sequences evolve independently.

Using *D. melanogaster* as an out-group species, the relative rates of molecular evolution were calculated for two of the proteins listed in Figure S2, Cdk1 and Moe, in mosquitoes. The χ^2^ test statistic based on Tajima's test [124] shows significant p values for Moe evolution between *A. aegypti-A. gambiae* and *C. quinquefasciatus*-*A. gambiae*, but not between *A. aegypti*-*C. quinquefasciatus*. This indicates that Moe has a differential evolutionary rate between Anophilinae and Culicinae mosquitoes but may have a similar evolutionary rate within Culicinae. Given that Moe functions in a variety of developmental processes (Figure S3, [125]), selective pressures might act on any of these processes.

#### Repetitive codon sequences of developmental genes

Developmental genes, in general, are known to be enriched with repetitive sequences [126], [127]. In particular, genes involved in organ development were previously identified [128] as one of major gene categories that contain repetitive sequences within coding regions. Very little is known about the function and evolution of repeat motifs of developmental genes. Repetitive regions of several developmental proteins are thought to be the cause of several neurodegenerative diseases in humans [126], [129]. In insects, the well described *opa* and *opa*-like repeats are found in essential developmental proteins [130]. These typically encode a stretch of up to approximately 30 glutamines, with interspersed histidine residues.

The protein sequences encoded by developmental genes surveyed in this investigation were analyzed. Analyses revealed that these genes encode proteins containing numerous repeats of amino acid residues of which repetition of serine residues was consistently predominant in each of the three mosquitoes (Table 5). Whether these repetitions are products of replication slippage of these genes or results of natural selection was not determined. However, a previous investigation on serine homopolymers in human proteins suggested that these structures are primarily shaped by natural selection forces but not by replication slippage. An abundant number of simple sequence repeats within the coding sequences of mosquito developmental genes was identified (Figure S4). The majority of these repeats represent codon repeats in these genes, and some also correspond to codon pair repeats (data not shown). Whether these repetitions have a functional impact on mosquito development is not known but represents an interesting question for further research.

#### miRNAs binding sites in orthologous developmental genes

miRNAs are short non-coding RNAs of ∼22 bases that post-transcriptionally regulate gene expression through binding to the 3′-untranslated regions (UTR) of target gene mRNAs. miRNA interactions with the network of protein-coding genes are believed to confer robustness to developmental genetic programs in animals [131]. In recent years, increasing evidence suggests that miRNAs are crucial regulators of development [131], [132], [133], [134]. For example, in *D. melanogaster*, miRNAs regulate a variety of developmental processes such as apoptosis, cell division, germline stem cell differentiation, oogenesis, and neural development, including olfactory development (reviewed by [135], [136]). A number of developmental genes have been experimentally verified as miRNA targets in flies. For example, *miR-315* and *miR-8* regulate components of the Wg pathway, while *miR-1* and *miR-7* regulate N pathway components during fly development (reviewed by [136]).

miRNA genes have been identified in *A. aegypti*, *C. quinquefasciatus*, and *A. gambiae* [137]. The number of individual copies of miRNA genes vary among the three mosquitoes (Figure S5). A few of these miRNA genes have been studied in the context of development. For example, in *A. aegypti*, miR-275 functions during egg development [72]. Developmental stage specific expression patterns of miRNA genes were also observed in *A. aegypti* [116] as well as in *A. stephensi* [138]. To better understand the functional role of miRNAs in mosquito development, comparative analysis of one-to-one orthologous developmental genes among *D. melanogaster*, *A. aegypti*, and *A. gambiae* that are predicted (computationally) as targets of miRNAs was performed. The *C. quinquefasciatus* targets have not yet been annotated and were therefore not included in this analysis. As single miRNAs can potentially regulate multiple targets, miRNAs with multiple binding sites in the developmental genes of these mosquitoes were curated.

Based on the rank order of the number of miR developmental target genes in each species, it was found that the repertoire of miRNA binding sites may be species-specific. The number of predicted developmental gene targets of various miRNAs varies within each species (Figure 6). The top 10 ranking miRNAs that are predicted as major regulators of developmental genes in mosquitoes and *Drosophila* are listed in Table 6. This empirical comparative analysis of predicted miRNA targets suggests that developmental regulation of miRNAs may have evolutionary signatures that are specific to each species. Besides variation in number of developmental genes as potential targets, the number of copies of miRNA genes also vary among the three mosquitoes (Figure S5). The temporal and spatial expression of the cognate miRNAs in these mosquitoes may have therefore diverged.

**Table 6:** Top 10 ranking miRNAs predicted as major regulators of developmental genes in mosquitoes and *Drosophila*.

Rank #	Aae	Aga	Dmel
**1**	*miR-9a*	*miR-34*	*miR-14*
**2**	*miR-124*	*miR-125*	*miR-92b*
**3**	*miR-10*	*miR-133*	*let-7*
**4**	*miR-263*	*miR-iab-4*	*miR-124*
**5**	*bantam*	*bantam*	*miR-210*
**6**	*miR-287*	*miR-92b*	*miR-283*
**7**	*miR-306*	*miR-9a*	*miR-305*
**8**	*miR-6*	*miR-124*	*miR-4*
**9**	*miR-14*	*miR-307*	*miR-5*
**10**	*miR-278*	*miR-1008*	*miR-8*

The rank order of miRNAs with the greatest number of predicted developmental gene targets varies in the *D. melanogaster* and mosquito genomes [137]. Ranks are reported from the highest (1) to lowest (10) predicted number of matches.

### Summation and Future Directions

We have made great advances in understanding developmental genetics in *D. melanogaster*, but comparatively little is known about the genetic basis for development in mosquitoes. Here, a comparative genomic approach was used to investigate developmental genetic changes that may underlie basic biological differences between *D. melanogaster* and vector mosquitoes, as well as between different mosquito species. As anticipated, although *Drosophila* developmental genes are largely very well conserved in vector mosquitoes (Table S1), several key regulators of fly development were not identified in one or more mosquito species (Tables 1, 2, 3, 4). Consideration of the known effects of loss-of-function mutation of such genes in *Drosophila*, as discussed throughout the text, may provide insight into the evolution of mosquito development.

It is of course difficult to know if the inability to identify a given gene truly reflects the absence of the gene in mosquitoes, or whether the gene could not be identified as a result of significant divergence from the *D. melanogaster* sequence used as the basis for the assignment. For example, Zhou *et al.* [73] indicated that they were not able to find orthologs for the IAP antagonists *grim*, *reaper*, and *hid* because of extensive sequence divergence. Michelob_X, believed to be the missing IAP antagonist, was identified through a customized searching strategy involving a motif search program. Although such customized motif searches for individual genes were not employed here due to the breadth of this investigation, in cases where genes were apparently missing, similarity searches were performed using the gene and protein sequences of non-*Drosophila* insect species orthologs (see Methods for details). However, in many cases, genes that were not identified in mosquitoes (Tables 1, 2, 3, 4) were previously reported to be missing in one or more other insect species. As discussed above, the absences of *zen*, *swa*, *grim*, *reaper*, *hid*, *grk*, *scw*, *bcd*, *sisA*, and *tra*, have been noted in other insects. However, some of the gene absences noted in mosquitoes were more surprising. For example, *argos*, which has been identified in other insects [125], was not identified in any of the mosquito genomes. In *D. melanogaster*, Argos, a negative regulator of EGF signaling [38], is critical for a number of developmental processes, such as wing, eye, haltere, genital, and nervous system development [125]. Furthermore, the absence of *Dad* in all three mosquitoes is interesting given that it has been identified in a number of insect species [125]. *Dad* encodes an anti-SMAD that functions in a variety of processes, such as digestive tract, renal tubule, and neural development in *D. melanogaster* [125].

A number of the lineage specific gene absences noted in this investigation (Tables 1, 2, 3, 4) were also unanticipated. For example, the inability to identify any *Pax3/7* orthologs *(prd, gsb, gsb-n)* in *A. aegypti* is unusual, as we are unaware of other insects that lack a *Pax3/7* gene. Prd, Gsb, and Gsb-n function in a variety of developmental process in *D. melanogaster*, perhaps most notably segmentation and neurogenesis, where their functions have been documented [125]. Furthermore, the inability to identify any *comm* gene in *A. gambiae* is surprising given the critical role that this gene plays in *D. melanogaster* embryonic ventral nerve cord development (reviewed in [59]). However, recent functional analyses suggest that the regulation of nerve cord development differs between mosquitoes and *D. melanogaster* ([18]; Haugen et al., submitted). Given these results, it will be interesting to functionally study the regulation of nerve cord development in *A. gambiae*, and also to functionally assess the roles of the *A. aegypti* and *C. quinquefasciatus comm* genes. Finally, as noted above, given the conservation of the FGF signaling pathway across many vertebrate and invertebrate species [40], our inability to identify any orthologs of the three known fly FGF ligands in *A. aegypti* is peculiar.

In several instances, with respect to *D. melanogaster*, an increased number of copies of particular developmental genes was observed in mosquitoes (Tables 1, 2, 3, 4). Some of the most striking examples include expansions of: i) *fz* (four in *A. aegypti*, three in *C. quinquefasciatus*, and two in *A. gambiae*), ii) *aub* (seven in *A. aegypti* and *C. quinquefasciatus*, and iii) several larval cuticle genes in *A. aegypti* (15 copies of *Ccp84Ad*; 10 copies of *CG7203*; 6 copies of *Cpr30B*; 9 copies each of *Cpr30F*, *Cpr65Eb*, and *Lcp65Ac*). Studying the function of these developmental genes is of great importance. As discussed by Patel and Prince [139], once duplicated, gene pairs can take on separable genetic functions in developing organisms. This can occur through changes in the coding region that lead to proteins with distinct biochemical functions. Furthermore, the duplicated genes may acquire different components of the original gene's enhancer/suppressor elements, resulting in distinct developmental expression patterns. Alternatively, changes in expression patterns of the two genes can arise from mutations in their enhancers. Exon shuffling, the generation of alternative transcripts, and evolution of novel enhancer elements can also occur once the gene has duplicated. The processes of duplication and divergence can occur multiple times, producing gene families of interest to evolutionary developmental biologists. Hox gene family evolution across all metazoans has been particularly well studied, and such detailed analyses of Hox genes have provided insight into the evolution of developmental processes [139]. Detailed functional studies of duplicated and expanded developmental genes in the three mosquito genomes will similarly enhance our understanding of the evolution of developmental processes in dipterans.

In conclusion, this study provides a resource for those who wish to pursue developmental genetic analyses in mosquitoes. The results of this study will also promote the design and refinement of functional analysis experiments. This investigation suggests that analysis of developmental processes regulated by Wnt/Fz, Notch, and FGF signaling may be of interest, as absences and gains of components of these signaling pathways were noted (Table 1, Figures 1, 2, 3). Furthermore, these genome wide comparisons indicate that functional analysis of segmentation, germline development, apoptosis (Table 2, Figure 4), salivary gland development (Table 3, Figure S2), head development (Table 3), cuticular development (Table 3, Figure 5), egg diapause (Table 4), and developmental transcripts targeted by mosquito miRNAs (Table 6, Fig. 6, Figure S5) may prove to be highly interesting.

## Methods

### Orthology assignments

Developmental genes of *D. melanogaster* were chosen based on Gene Ontology annotation in FlyBase (http://flybase.net) [125], through information posted in Interactive Fly (http://www.sdbonline.org/fly/aimain/1aahome.htm) [35], through literature surveys, and in reference to the genes selected for a recently published comprehensive survey of developmental genes in *A. pisum* [25]. Orthology calls were prepared with the aid of several databases: Biomart (http://www.biomart.org/biomart/martview/) [33], Vectorbase (http://www.vectorbase.org/) [34], Flybase (http://flybase.org/) [125], OrthoDB (http://cegg.unige.ch/orthodb4) [140], InParanoid (http://inparanoid.cgb.ki.se/) [141], and (miRBase http://www.mirbase.org/) [137]. Splice variants were excluded in this study. For cases in which no ortholog was identified or in which discrepancies between databases were observed, reciprocal BLAST [142] searches (tblastx and tblastn) were used to identify orthologs or confirm orthology statuses. Such BLAST searches were performed with the *Drosophila* gene, and when available, orthologs from non-*Drosophila* species, or with the mosquito ortholog(s) identified in databases. If multiple hits were identified through BLAST searches, tblastx and tblastn results were assessed for hits common to both searches, which helped to eliminate false positives resulting from codon bias. Final ortholog assignments were made through analysis of ClustalW [143] alignments and by construction of Neighbor joining (NJ) phylogenetic trees using Molecular Evolutionary Genetics Analysis (MEGA) Version 4 [144]. Bootstrap analysis of phylogeny was performed with 1000 replicates. The Poisson correction model was used as a distance measure. Uniform rates among sites and homogenous substitution patterns between lineages were assumed.

### Coefficient of evolutionary differentiation estimate

The coefficient of evolutionary differentiation was estimated according to the methods of Zuckerkandl and Pauling [145] implemented in MEGA4 [144]. All results are based on the pairwise analysis of 15 sequences, including three mosquito and 12 fruit fly species. Genes analyzed were preselected based on one-to-one orthologies among the 15 insects species as annotated by hierarchical ortholog clustering by OrthoDB. Analyses were conducted using the Poisson correction as distance as described in [146]. All positions containing gaps and missing data were eliminated from the dataset (complete deletion option). Uniform rates among sites and homogenous substitution patterns between lineages were assumed.

### Simple sequence repeat (SSR) identification

The gene sequences were subjected to SciRoKo software [147], a freely available SSR identification program (http://kofler.or.at/bioinformatics/SciRoKo/). The program was set to the default parameters (mismatch, fixed penalty) to extract both perfect and imperfect repeat sequences within each gene.

### Distribution of miRNA binding sites within developmental genes of mosquitoes

The microRNA genes and predicted targets were obtained from miRbase (http://www.mirbase.org/) [137] and MicroCosm Targets Version 5 (http://www.ebi.ac.uk/enright-srv/microcosm/htdocs/targets/v5/) respectively. Developmental genes with miR targets were identified from the downloaded target list using the ‘vlookup’ formula in Excel. The quantification and comparison of miR targets and miR genes were performed by Excel.

## Supporting Information

Table S1 **Survey of** ***D. melanogaster*** **developmental gene orthologs in** ***A. aegypti*** **,** ***C. quinquefasciatus*** **, and** ***A. gambiae*** **.** The associated gene names in *D. melanogaster*, gene identification numbers for each species, and orthology types are indicated.
(XLSX)

Figure S1 **Evolutionary relationships of** ***Net*** **orthologs.** Phylogenetic relations of *NetA* and *NetB* genes among *D. melanogaster* and the three mosquito species (gene IDs are shown). The optimal tree of NetA sequences with the sum of branch length = 1.253 and that of NetB with sum of branch length = 3.284 are shown. The percentage values of replicate trees in which the associated taxa clustered together following bootstrap testing (1000 replicates) are shown next to the branches. The tree is drawn to scale (shown below the tree), with branch lengths in the same units as those of the evolutionary distances used to infer the phylogeny. The distance scale is in units of the number of amino acid substitutions per site.
(TIF)

Figure S2 **Evolutionary differentiation of developmental genes.** Estimates of the coefficients of evolutionary differentiation for one-to-one developmental gene orthologs in the three mosquito and twelve *Drosophila* genomes are indicated. The estimates are based on amino acid substitutions per site. Known functions of these proteins in *D. melanogaster* [125] are also indicated. The results indicate that retaining a singleton copy of a gene in the mosquito and fruit fly genomes does not necessarily confer any selection constraint on the sequence.
(TIF)

Figure S3 **Estimates of evolutionary rates for the** ***cdk1*** **and** ***moe*** **genes in mosquitoes compared to** ***D. melanogaster*** **.** The rate is estimated between pair-wise comparisons of mosquito genes with the *D. melanogaster* ortholog as the out-group sequence. The number of identical sites and sites that are divergent among the genes are shown under the respective headings. The number of sites that are uniquely evolved in mosquito genes and *Drosophila* genes are shown in the next two columns. The χ^2^ test statistic represents a statistical significance measure whether to reject the null hypothesis (that the evolutionary rates are the same between the two mosquitoes). A P value <0.05 is considered significant and suggests different rates of evolution between mosquitoes.
(TIF)

Figure S4 **Simple sequence repeats in developmental genes.** An abundant number of simple sequence repeats (one to six bp motif repeats) are found within codon sequences of developmental genes (one-to-one orthologs) in the three mosquitoes. Numbers reported in the counts column correspond to the total number of each type of repeat observed in the developmental genes studied (listed in Table S1) for each of the three mosquito species. Numbers in the average length column correspond to the average length of the repeat in nucleotides. Some repeats are not perfect, as illustrated by the average numbers of mismatches reported in the column at right. These data indicate that the total number of repeats in developmental genes and average length of repeats vary among the three species.
(TIF)

Figure S5 **Variation in the number of miRNA genes among the three mosquito genomes.** Some miR genes are present in multiple copies in one or more mosquito species. Numerical values (in parentheses) correspond to the total number of mIR copies in the indicated mosquito species. Results are reported only for species in which multiple copies of a miR gene exist.
(TIF)
