# Supplementary figures and images for: Comparative Genomic Analysis of Drosophila melanogaster and Vector Mosquito Developmental Genes
Source: PLoS One. 2011 Jul 6;6(7):e21504. doi: 10.1371/journal.pone.0021504 (PMC3130749; doi:10.1371/journal.pone.0021504)

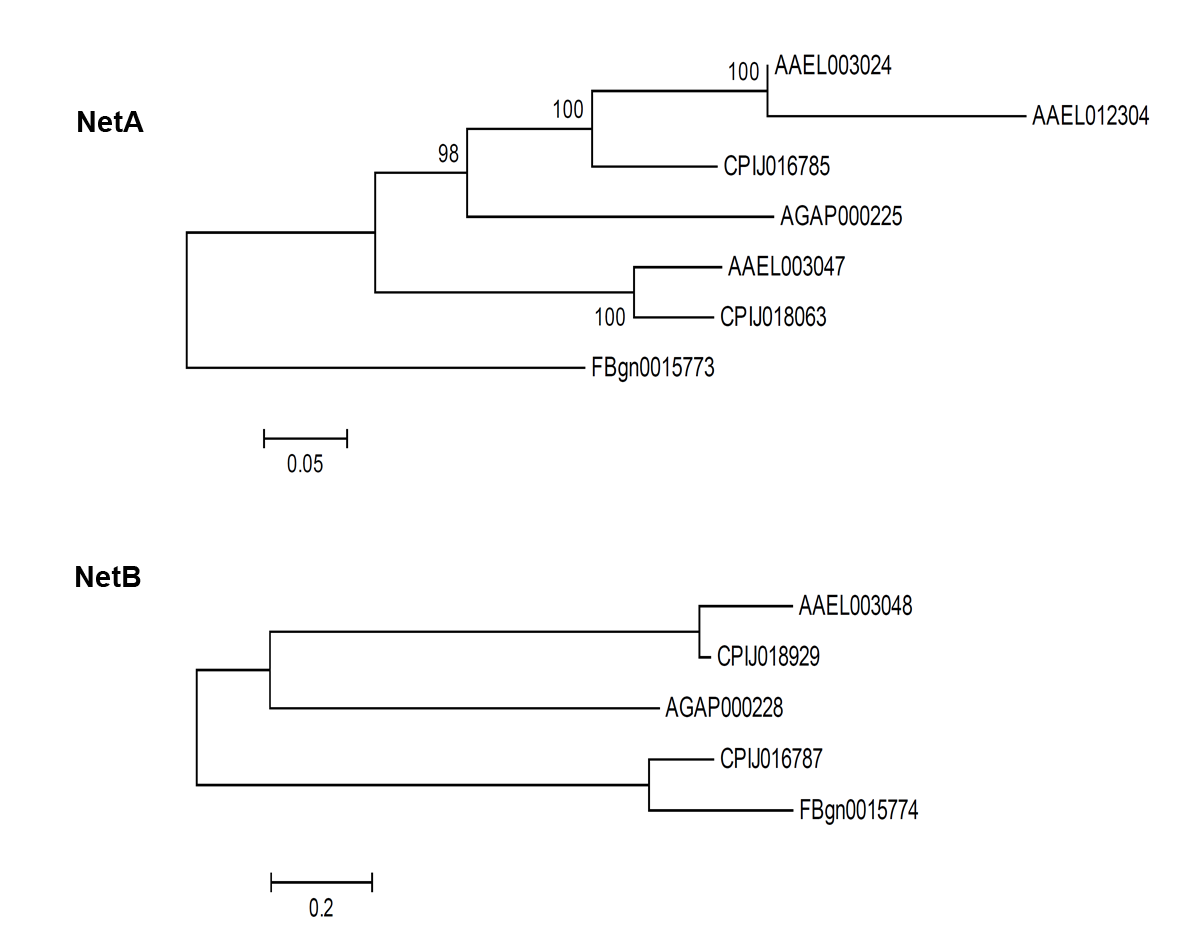

Supplement: Figure S1 — Evolutionary relationships of Net orthologs. Phylogenetic relations of NetA and NetB genes among D. melanogaster and the three mosquito species (gene IDs are shown). The optimal tree of NetA sequences with the sum of branch length = 1.253 and that of NetB with sum of branch length = 3.284 are shown. The percentage values of replicate trees in which the associated taxa clustered together following bootstrap testing (1000 replicates) are shown next to the branches. The tree is drawn to scale (shown below the tree), with branch lengths in the same units as those of the evolutionary distances used to infer the phylogeny. The distance scale is in units of the number of amino acid substitutions per site. (TIF) [file pone.0021504.s002.tif]

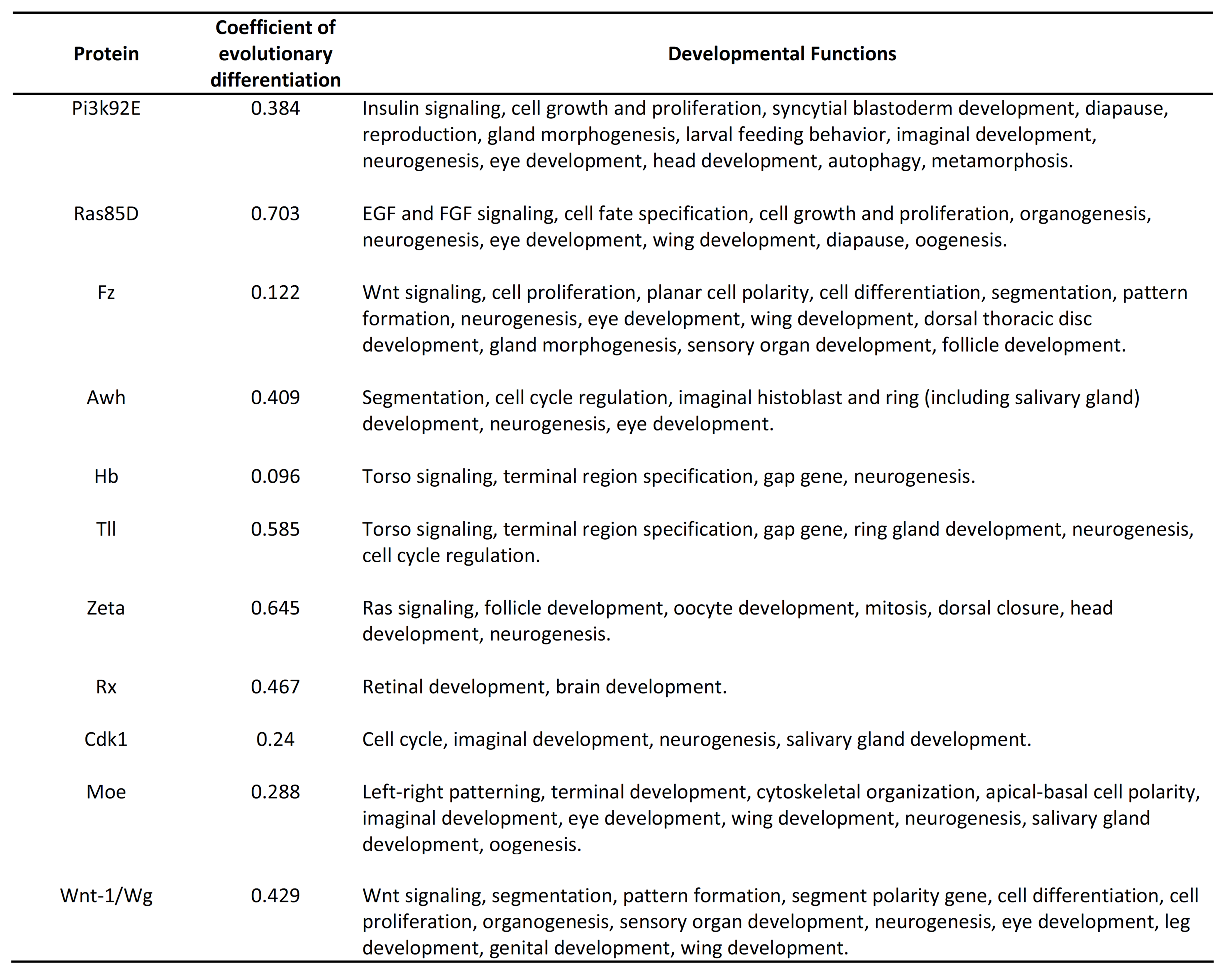

Supplement: Figure S2 — Evolutionary differentiation of developmental genes. Estimates of the coefficients of evolutionary differentiation for one-to-one developmental gene orthologs in the three mosquito and twelve Drosophila genomes are indicated. The estimates are based on amino acid substitutions per site. Known functions of these proteins in D. melanogaster [125] are also indicated. The results indicate that retaining a singleton copy of a gene in the mosquito and fruit fly genomes does not necessarily confer any selection constraint on the sequence. (TIF) [file pone.0021504.s003.tif]

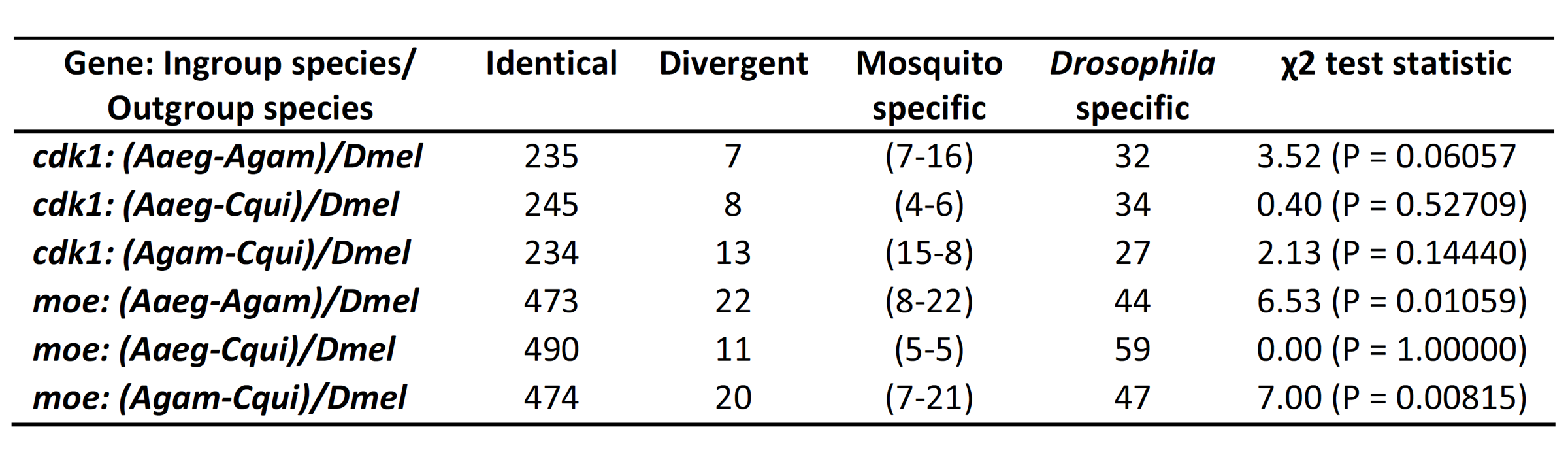

Supplement: Figure S3 — Estimates of evolutionary rates for the cdk1 and moe genes in mosquitoes compared to D. melanogaster . The rate is estimated between pair-wise comparisons of mosquito genes with the D. melanogaster ortholog as the out-group sequence. The number of identical sites and sites that are divergent among the genes are shown under the respective headings. The number of sites that are uniquely evolved in mosquito genes and Drosophila genes are shown in the next two columns. The χ2 test statistic represents a statistical significance measure whether to reject the null hypothesis (that the evolutionary rates are the same between the two mosquitoes). A P value <0.05 is considered significant and suggests different rates of evolution between mosquitoes. (TIF) [file pone.0021504.s004.tif]

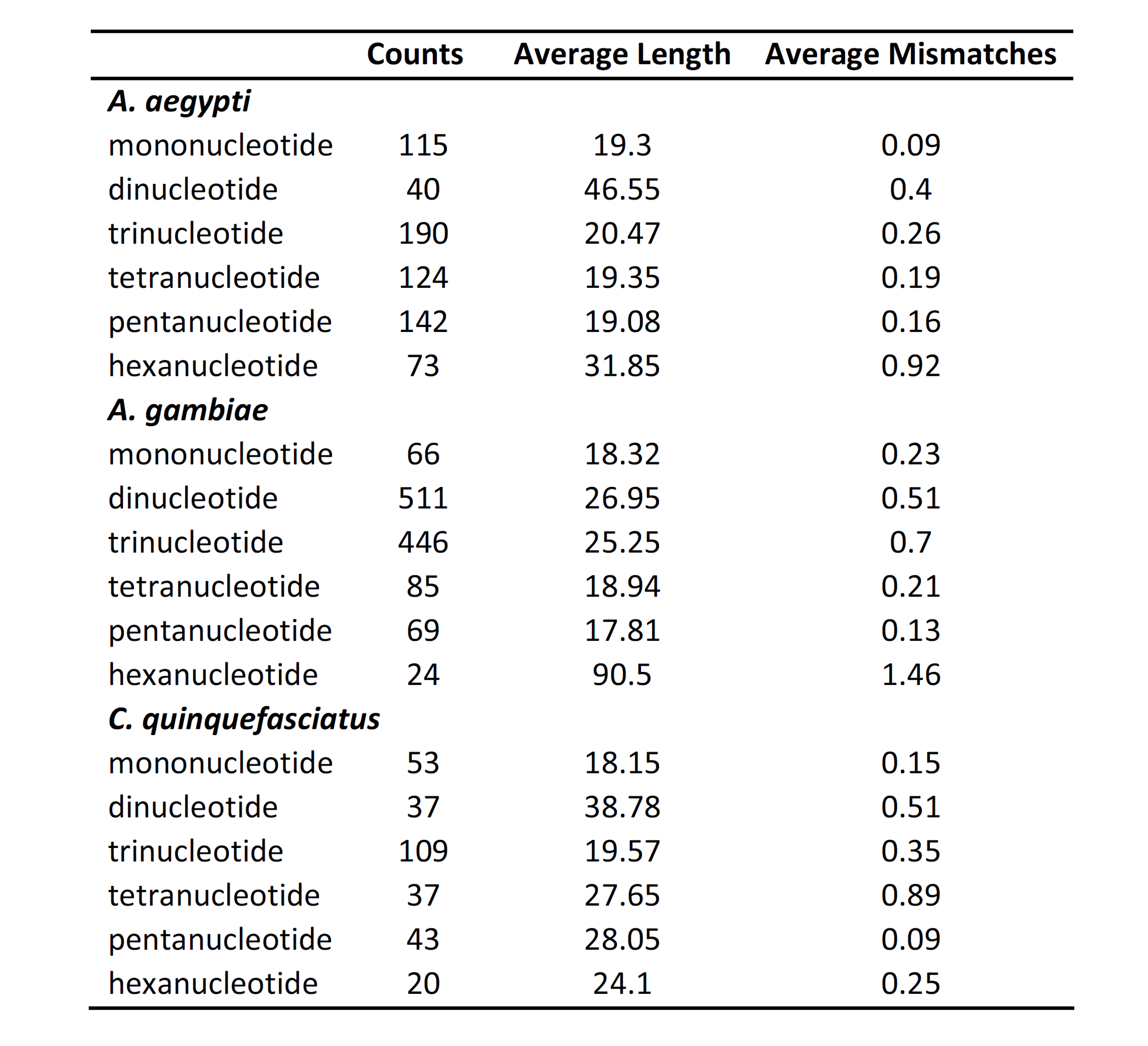

Supplement: Figure S4 — Simple sequence repeats in developmental genes. An abundant number of simple sequence repeats (one to six bp motif repeats) are found within codon sequences of developmental genes (one-to-one orthologs) in the three mosquitoes. Numbers reported in the counts column correspond to the total number of each type of repeat observed in the developmental genes studied (listed in Table S1) for each of the three mosquito species. Numbers in the average length column correspond to the average length of the repeat in nucleotides. Some repeats are not perfect, as illustrated by the average numbers of mismatches reported in the column at right. These data indicate that the total number of repeats in developmental genes and average length of repeats vary among the three species. (TIF) [file pone.0021504.s005.tif]

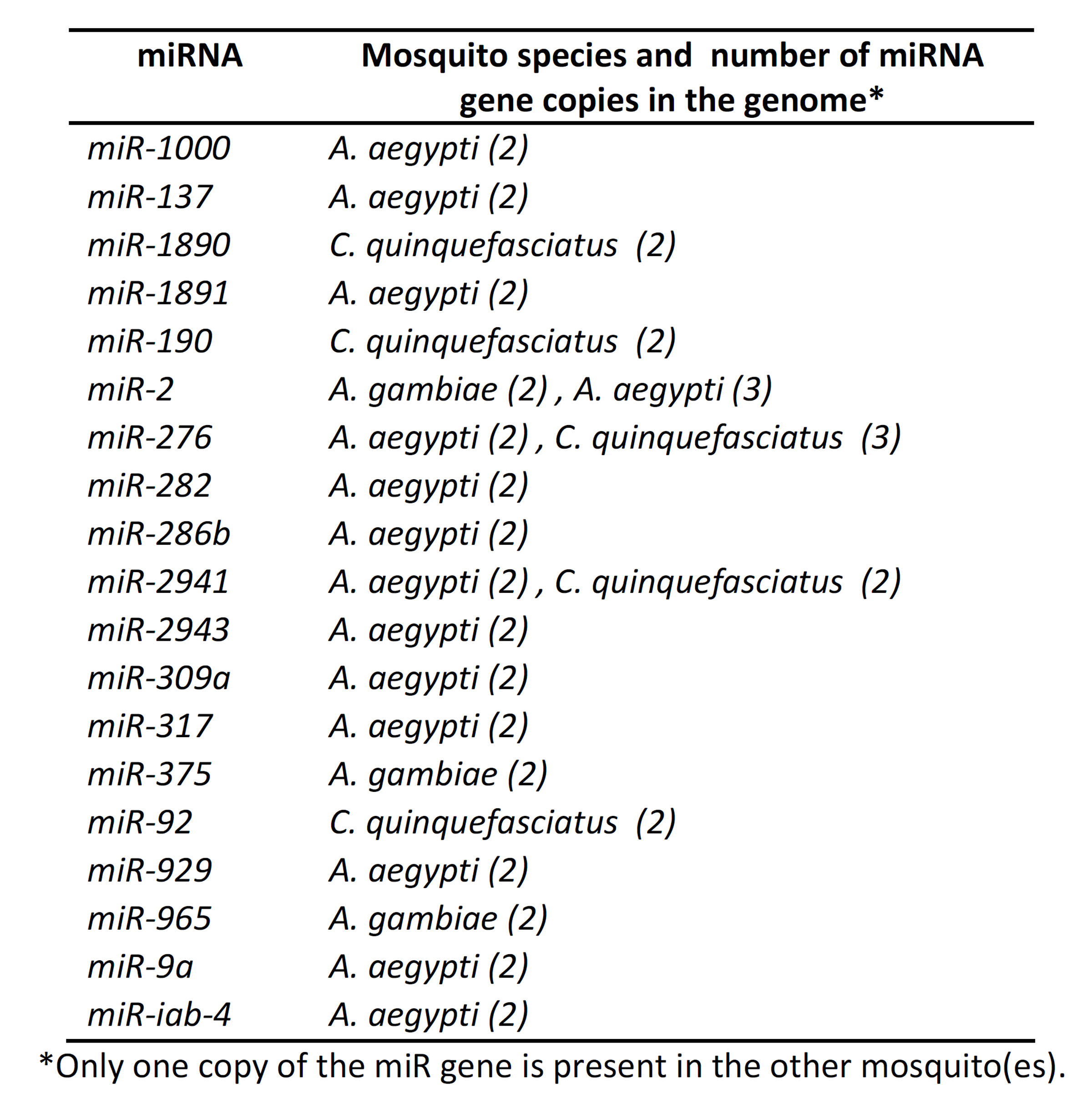

Supplement: Figure S5 — Variation in the number of miRNA genes among the three mosquito genomes. Some miR genes are present in multiple copies in one or more mosquito species. Numerical values (in parentheses) correspond to the total number of mIR copies in the indicated mosquito species. Results are reported only for species in which multiple copies of a miR gene exist. (TIF) [file pone.0021504.s006.tif]
